# Screening Mycobacterium tuberculosis Secreted Proteins Identifies Mpt64 as a Eukaryotic Membrane-Binding Bacterial Effector

**DOI:** 10.1128/mSphere.00354-19

**Published:** 2019-06-05

**Authors:** Chelsea E. Stamm, Breanna L. Pasko, Sujittra Chaisavaneeyakorn, Luis H. Franco, Vidhya R. Nair, Bethany A. Weigele, Neal M. Alto, Michael U. Shiloh

**Affiliations:** a Department of Internal Medicine, University of Texas Southwestern Medical Center, Dallas, Texas, USA; b Department of Microbiology, University of Texas Southwestern Medical Center, Dallas, Texas, USA; c Department of Pediatrics, University of Texas Southwestern Medical Center, Dallas, Texas, USA; d Center for Autophagy Research, University of Texas Southwestern Medical Center, Dallas, Texas, USA; University of Kentucky

**Keywords:** *Mycobacterium tuberculosis*, effector functions, pathogenesis

## Abstract

Mycobacterium tuberculosis (Mtb), the causative agent of tuberculosis, is one of the most successful human pathogens. One reason for its success is that Mtb can reside within host macrophages, a cell type that normally functions to phagocytose and destroy infectious bacteria. However, Mtb is able to evade macrophage defenses in order to survive for prolonged periods of time. Many intracellular pathogens secrete virulence factors targeting host membranes and organelles to remodel their intracellular environmental niche. We hypothesized that Mtb secreted proteins that target host membranes are vital for Mtb to adapt to and manipulate the host environment for survival. Thus, we characterized 200 secreted proteins from Mtb for their ability to associate with eukaryotic membranes using a unique temperature-sensitive yeast screen and to manipulate host trafficking pathways using a modified inducible secretion screen. We identified five Mtb secreted proteins that both associated with eukaryotic membranes and altered the host secretory pathway. One of these secreted proteins, Mpt64, localized to the endoplasmic reticulum during Mtb infection of murine and human macrophages and impaired the unfolded protein response in macrophages. These data highlight the importance of secreted proteins in Mtb pathogenesis and provide a basis for further investigation into their molecular mechanisms.

**IMPORTANCE** Advances have been made to identify secreted proteins of Mycobacterium tuberculosis during animal infections. These data, combined with transposon screens identifying genes important for M. tuberculosis virulence, have generated a vast resource of potential M. tuberculosis virulence proteins. However, the function of many of these proteins in M. tuberculosis pathogenesis remains elusive. We have integrated three cell biological screens to characterize nearly 200 M. tuberculosis secreted proteins for eukaryotic membrane binding, host subcellular localization, and interactions with host vesicular trafficking. In addition, we observed the localization of one secreted protein, Mpt64, to the endoplasmic reticulum (ER) during M. tuberculosis infection of macrophages. Interestingly, although Mpt64 is exported by the Sec pathway, its delivery into host cells was dependent upon the action of the type VII secretion system. Finally, we observed that Mpt64 impairs the ER-mediated unfolded protein response in macrophages.

## Abstract (precis)

Advances have been made to identify secreted proteins of Mycobacterium tuberculosis during animal infections. These data, combined with transposon screens identifying genes important for M. tuberculosis virulence, have generated a vast resource of potential M. tuberculosis virulence proteins. However, the function of many of these proteins in M. tuberculosis pathogenesis remains elusive. We have integrated three cell biological screens to characterize nearly 200 M. tuberculosis secreted proteins for eukaryotic membrane binding, host subcellular localization, and interactions with host vesicular trafficking. In addition, we observed the localization of one secreted protein, Mpt64, to the endoplasmic reticulum (ER) during M. tuberculosis infection of macrophages. Interestingly, although Mpt64 is exported by the Sec pathway, its delivery into host cells was dependent upon the action of the type VII secretion system. Finally, we observed that Mpt64 impairs the ER-mediated unfolded protein response in macrophages.

## INTRODUCTION

Tuberculosis caused by Mycobacterium tuberculosis (Mtb) is a persistent, global epidemic. While the number of deaths due to Mtb fell below 2 million in 2015, there were more than 9 million new cases of tuberculosis (1), and the incidence of multidrug-resistant tuberculosis is increasing (1), highlighting the need for new antituberculosis therapies. In addition, the only currently available vaccine, Mycobacterium bovis bacillus Calmette-Guérin (BCG), is ineffective in preventing pulmonary tuberculosis infection (2). Thus, understanding the intracellular survival mechanisms employed by Mtb is vital to developing new antituberculosis treatments and vaccines.

Macrophages, phagocytic innate immune cells that are generally competent for bacterial killing, represent the primary intracellular niche for Mtb. Some of the antimicrobial mechanisms utilized by macrophages include acidification of the phagosome, production of reactive oxygen and nitrogen species, fusion of lysosomes to bacteria containing phagosomes, and autophagy (3–6). However, despite these robust defenses, Mtb survives inside macrophages during its infectious life cycle. To facilitate its survival, Mtb resists macrophage defenses, either by directly protecting the bacterial cell from damage (7–9) or by modulating the macrophage’s ability to shuttle the bacteria through the traditional phagolysosomal maturation process (10). In that way, Mtb prevents its intracellular compartment from acidifying (11) and fusing (12) with the destructive lysosome. Genetic studies have identified several Mtb genes important for remodeling host membrane trafficking (13–15). For example, Mtb *Rv3310* encodes SapM, a secreted acid phosphatase (16) that converts phosphatidylinositol 3-phosphate (PI3P) to phosphatidylinositol. Loss of PI3P from the phagosome membrane is sufficient to prevent fusion of phagosomes with late endosomes (17, 18). Importantly, many genes reported to be important for Mtb survival inside macrophages remain uncharacterized (13, 14, 19), and the manipulation of the host cell by Mtb remains poorly understood.

The problem of intracellular survival faced by Mtb is also shared by other bacterial pathogens, and many of these organisms utilize specialized secretion systems to deliver molecules into the host cell to establish a unique intracellular niche (20). For example, some Gram-negative pathogens use needle-like machines encoded by type III secretion systems (TTSS) that span the bacterial and host cell membranes to inject protein cargo into the host (21–24). Another specialized secretion machine called a type IV secretion system is found in Gram-positive and Gram-negative bacteria and can be used by many human and plant pathogens such as Legionella pneumophila, Coxiella burnetii, and Agrobacterium tumefaciens to transport effector proteins that promote bacterial survival (25, 26). Finally, Mtb contains genes that encodes multiple type VII secretion systems, discussed below, that are important in pathogenesis (27–29).

In addition to the type VII secretion systems, Mtb contains genes that encode components of the conserved general secretion system (Sec) and the twin-arginine translocation pathway (Tat), both of which are essential for growth in Mtb (30). The Mtb Sec system is the primary route for protein export, and Sec substrates have both housekeeping and virulence activities (30, 31). For example, Mtb lacking *lspA*, which encodes the signal peptidase that cleaves the signal sequence of Sec-dependent lipoproteins, is attenuated in infection models (32), and Sec-dependent lipoproteins such as LprG (33, 34) and LpqH (35) are also important for Mtb virulence. Mtb also contains genes that encode components of a second, accessory Sec system, SecA2, that is required for Mtb growth in macrophages, possibly by dampening the host immune response (36). Substrates of the accessory SecA2 system such as the protein kinase PknG and the esterase LipO are important for Mtb virulence by contributing to phagosome maturation arrest (PMA) (13, 18, 37, 38). Because the Tat system is essential in Mtb (39), it has not been studied in pathogenesis models, though at least one virulence factor, phospholipase C is known to be a substrate of the Tat system (40). Finally, as noted above, Mtb contains genes that encode multiple type VII (also called ESX) secretion systems (28). ESX-3 is essential for growth *in vitro* (41), while the ESX-1 and ESX-5 systems are required for Mtb virulence in macrophages and mouse models of infection (27, 42, 43). Though many of the ESX substrates have been identified through proteomics (44), their activities are mostly unknown, though some ESX substrates such as ESAT-6 (EsxA), CFP-10 (EsxB), and PE/PPE proteins may modulate the host immune response (45–48).

Effectors are proteins that promote bacterial survival by manipulating vital cellular processes, including signal transduction and vesicular trafficking, and the cytoskeleton (49–51). Like the secretion systems themselves, the repertoire of effectors expressed by each pathogen can differ, adapted specifically for each unique life cycle. However, host membranes are a major common target for effectors. For example, SifA from Salmonella enterica serovar Typhimurium (*S.* Typhimurium) is prenylated inside the host cell and localizes to the *Salmonella*-containing vacuole (SCV) (52). SifA recruits lysosomes to maintain SCV membrane integrity, and its membrane interaction is vital to *Salmonella* pathogenicity (52, 53). The L. pneumophila type IV secreted effector SidM is anchored to the *Legionella*-containing vacuole and disrupts host vesicle trafficking by sequestering and modifying Rab1 (54). Some effectors can also function by directly modifying membranes such as IpgD from Shigella flexneri that hydrolyzes phosphatidylinositol 4,5-bisphosphate [PI(4,5)P_2_] to phosphatidylinositol 5-monophosphate (PI5P), leading to membrane blebbing and bacterial uptake into host cells (55). Thus, both host membranes themselves and membrane-dependent processes represent valuable targets for bacterial effectors (49, 51, 56) as we recently showed for a variety of bacterial pathogens (57). Because membrane processes are high-value targets of many bacterial effectors (57, 58) and Mtb has a large repertoire of secreted proteins of unknown function (59–63), we hypothesized that some of the Mtb secreted proteins are membrane-binding effectors with virulence activities.

To test our hypothesis, we generated a library of 200 secreted proteins from Mtb, tested whether they individually bound yeast membranes in a life-or-death assay, and characterized their ability to alter host protein secretion in an inducible secretion assay. We also determined the subcellular localization of membrane-binding proteins using fluorescence microscopy. By combining data from the cell biological screens, we identified five Mtb secreted proteins that localized to eukaryotic membranes and disrupted the host secretory pathway in a model system. One protein, Mpt64 (Rv1980c), localized to the endoplasmic reticulum (ER) during both heterologous expression in HeLa cells and Mtb infection of macrophages. Though Mpt64 is a Sec substrate, its access to the macrophage cytoplasm was dependent on the ESX-1 secretion system. Finally, Mpt64 alone was sufficient to partially inhibit the unfolded protein response (UPR), suggesting a possible role for Mpt64 in regulating the macrophage response to infection.

## RESULTS

### Categorization of putative effector-like proteins from Mtb.

Through the analysis of published data sets, we identified Mtb proteins that may function as secreted effectors (see Table S1 in the supplemental material). For simplicity, we define these putative effectors as mycobacterial secreted proteins (MSPs), as this encompasses proteins that may be secreted to the mycobacterial surface, into the exoproteome (i.e., the extracellular milieu), or delivered into the host cell (64–67). We used the following criteria to assemble a library of MSPs: (i) Mtb proteins identified via unbiased proteomic approaches that are either in the cell wall or the exoproteome (60–63, 68, 69), (ii) Mtb proteins known to be involved in manipulation of host vesicular trafficking pathways, such as ones that induce mammalian cell entry (MCE) (70–72) or phagosome maturation arrest (13, 14, 73), (iii) a subset of PE/PPE proteins and proteins related to those encoded by type VII (ESX-1) loci (42, 48, 74), and (iv) proteins involved in virulence, ranging from defined to unknown functions (19, 75–78). We then used Gateway recombination cloning to subclone MSPs from the freely available Mtb ORFome Gateway compatible library (BEI) into destination vectors for a variety of subsequent assays. The comprehensive list of MSPs is shown in Table S1.

10.1128/mSphere.00354-19.6 TABLE S1 Characterization of mycobacterial secreted proteins. Proteins are listed in order of their corresponding gene open reading frame (ORF). The tabs summarize proteins identified in the Ras rescue assay (Ras “hits”) or hGH release assay (hGH “hits”). The five overlapping proteins are highlighted in red text in the hGH “hits” tab. Download Table S1, XLSX file, 0.01 MB. Copyright © 2019 Stamm et al. 2019 Stamm et al. This content is distributed under the terms of the Creative Commons Attribution 4.0 International license.

### Mtb genes encode secreted proteins that interact with eukaryotic membranes.

**FIG 1:**
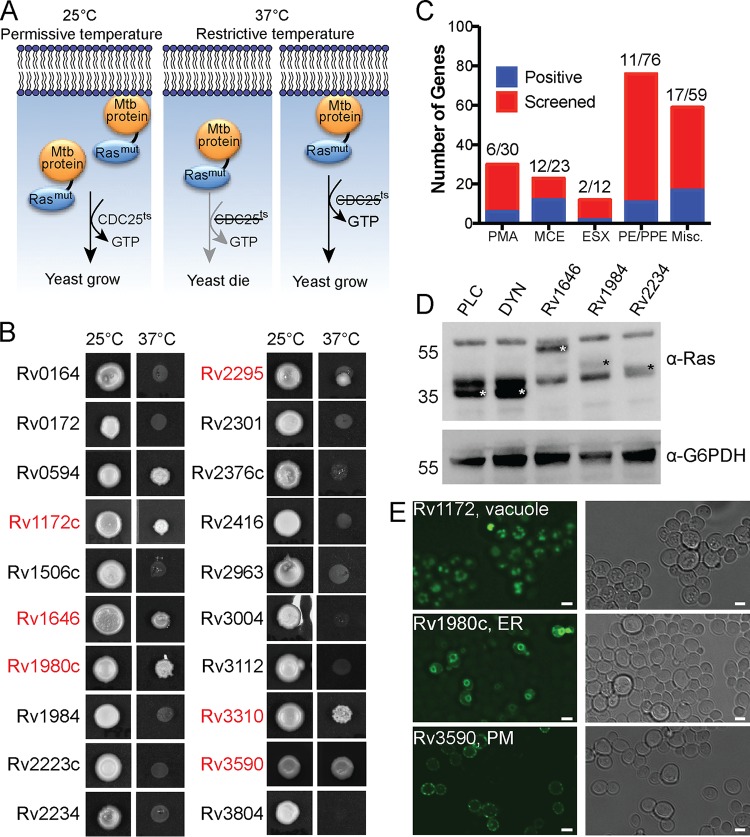
M. tuberculosis secreted proteins interact with yeast membranes. (A) Ras rescue assay schematic. (B) S. cerevisiae (cdc25^ts^) transformed with Mtb protein fusions to Ras^mut^, duplicate plated, and incubated for 48 to 72 h at the permissive (25°C) and restrictive (37°C) temperatures. Shown are representative images of 20 yeast strains from one experiment. The yeast strains that were rescued at the restrictive temperature are shown in red. (C) Summary results of the Ras rescue screen. (D) Western blot of lysates from yeast transformed with the indicated fusion proteins and probed with anti-Ras (α-Ras) or anti-G6PDH (α-G6PDH) antibodies. Ras^mut^ fusion proteins are marked by white or black asterisks. PLC (phospholipase C) and DYN (dynamin) are fusions to Ras^mut^ known to be membrane associated (PLC) or cytoplasmic (DYN). The results of one experiment of a total of three experiments are shown. (E) Representative fluorescence microscopy of S. cerevisiae (INVSc1) transformed with GFP-MSP fusion proteins. Images are representative of three independent experiments. PM, plasma membrane. Bars, 3 μm.

To identify membrane-binding Mtb proteins, we used a system that leverages the signal transduction of the essential Saccharomyces cerevisiae GTPase Ras to promote growth and division (79). Ras is lipidated at its CaaX box sequence that promotes its localization to the plasma membrane, where it can be activated by Cdc25, a guanine nucleotide exchange factor (79, 80). In a yeast strain with a temperature-sensitive *CDC25* allele, yeast can grow only at the permissive temperature (25°C), not the restrictive temperature (37°C), because Ras activation requires interaction with Cdc25. Heterologous expression of a nonlipidated, constitutively active Ras whose activity is independent of Cdc25 (mutant Ras [Ras^mut^]) can rescue yeast growth at the restrictive temperature when Ras is recruited to intracellular membranes by fusion to a membrane-binding protein (Fig. 1A). This system has been used to successfully identify membrane-binding effectors from Gram-negative pathogens (57). To identify membrane-localizing proteins from Mtb, we subcloned MSPs into a destination vector for yeast expression that generates an in-frame fusion of the MSP to Ras^mut^. We transformed S. cerevisiae *cdc25^ts^* (57, 79) individually with each of the 200 MSPs fused to Ras^mut^ and incubated them at both permissive and restrictive temperatures (Fig. 1B). Shown are 20 examples of yeast growth at 25°C and 37°C when expressing individual Ras^mut^-MSP fusion proteins. All strains grew when incubated at 25°C. However, at the restrictive temperature, only some yeast strains, indicated in red, were rescued. For example, we observed yeast growth rescue in yeast expressing a Ras^mut^ fusion to SapM (Rv3310), as expected due to its PI3P phosphatase activity (16, 18), but not for yeast expressing Ras^mut^ to the protein tyrosine phosphatase PtpA (Rv2234) (81, 82), which did not grow at 37°C (Fig. 1B). We identified 52 Mtb proteins that rescued S. cerevisiae *cdc25^ts^* growth at the restrictive temperature (Fig. 1B and C).

We confirmed expression of the Ras^mut^-MSP fusion proteins by Western blotting (Fig. 1D). In addition, we determined the membrane localization of each MSP by fluorescence microscopy of GFP-MSP fusion proteins in yeast (Fig. 1E). It has been established that Ras can function from membranes other than the plasma membrane (83, 84), and Ras^mut^ maintains this function (57). Thus, using fluorescence microscopy, we observed green fluorescent protein (GFP)-MSP fusion proteins localizing to distinct subcellular compartments, including vacuoles, ER, and plasma membrane (Fig. 1E). Together, these results show that 25% of the MSPs tested could associate with the membranes of a variety of organelles in S. cerevisiae.

### Subcellular localization of membrane-localizing MSPs.

**FIG 2:**
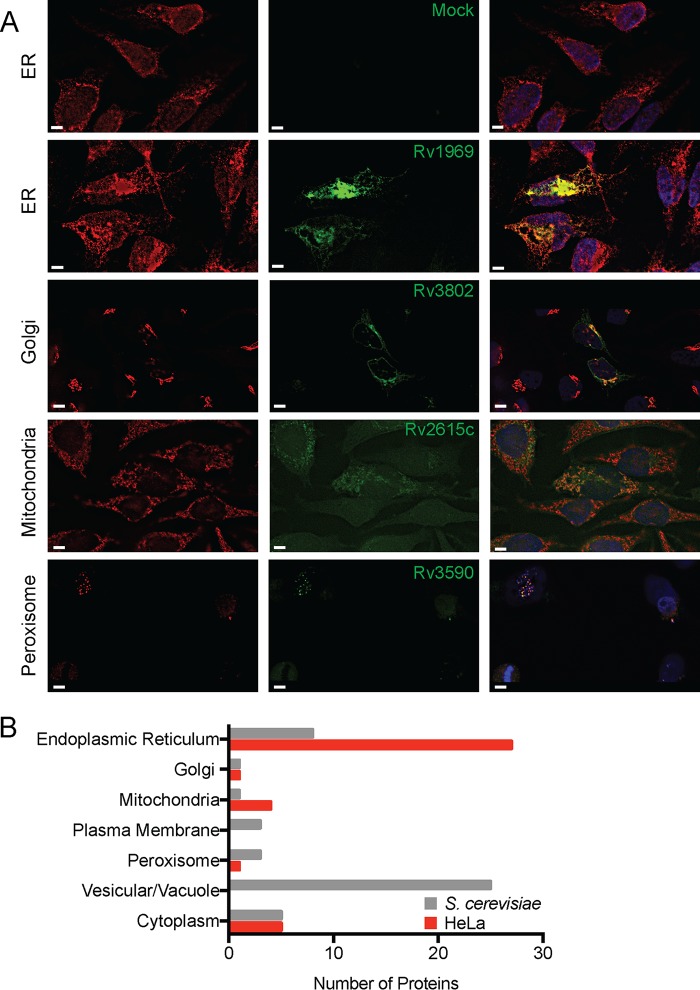
Host subcellular localization of membrane-binding MSP. (A) Fluorescent images of HeLa cells transfected with the indicated GFP-MSP fusion proteins (green) and stained with antibodies (red) to calreticulin (ER), GM-130 (Golgi), Tom20 (Mitochondria), or PMP70 (Peroxisomes). Images are representative of two independent experiments. Ten fields of about five cells each were observed for colocalization. Bars, 5 μm. (B) Comparison of the organelle localization of MSP expressed in yeast and HeLa cells.

While many cellular processes are conserved in eukaryotes, humans represent the primary natural host for Mtb. Therefore, to confirm that MSPs that rescued S. cerevisiae *cdc25^ts^* growth at 37°C also bound mammalian membranes and to determine their subcellular localization in human cells, we transiently transfected HeLa cells with vectors for constitutive expression of GFP-MSP fusion proteins and then used fluorescence microscopy with colocalization markers to identify the specific membrane to which each MSP localized (Fig. 2A). We identified GFP-MSP fusion proteins that localized to a variety of subcellular compartments, including the ER, Golgi apparatus, mitochondria, and peroxisomes (Fig. 2A and B and Table S1). The largest proportion of the GFP-MSP fusion proteins expressed in human cells colocalized with the ER marker calreticulin (Fig. 2B). We observed in yeast a similar number of GFP-Mtb fusion proteins that localized to compact, punctate structures that could not be definitively localized. Although there was only moderate overlap in the subcellular localization identified between yeast and HeLa cells (Fig. 2B), we were able to verify that the proteins identified by the Ras rescue assay are localized to membranous organelles in human cells.

### A subset of mycobacterial secreted proteins alter eukaryotic vesicular transport.

**FIG 3:**
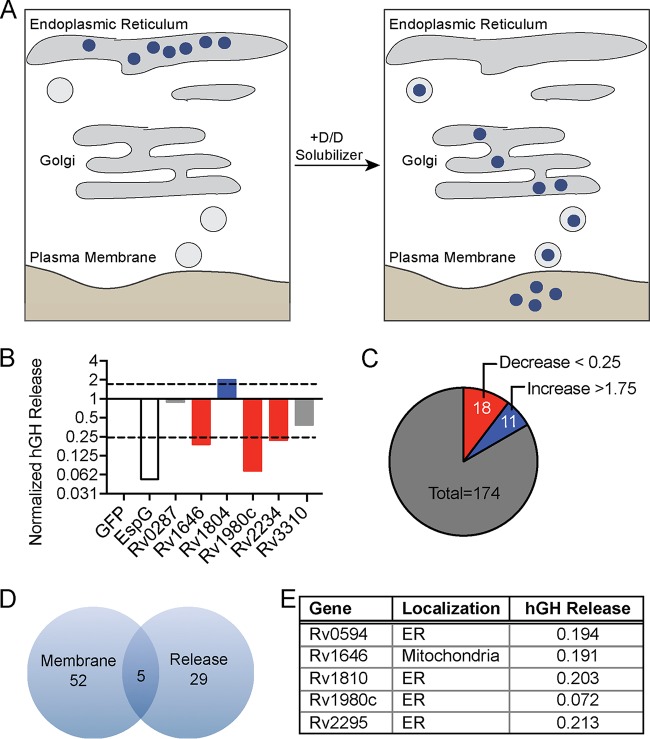
M. tuberculosis secreted proteins alter hGH secretion. (A) Inducible secretion assay schematic. (B) Supernatant hGH ELISA from HeLa cells transfected overnight with hGH-CAD and either GFP or GFP-MSP fusion proteins prior to the addition of drug to allow for hGH release. hGH release by GFP-MSP transfected cells was normalized to hGH release of cells transfected with GFP alone. hGH release was measured once for the entire group of GFP-MSP transfections. (C) Summary of results from inducible secretion screen. (D) Venn diagram of MSPs that are membrane localized, alter host secretion, or both. (E) Table summarizing the membrane localization and degree of hGH secretion in cells transfected with the five overlapping proteins from panel D.

Membrane-bound cargo is transported within the host cell and to the extracellular space in dynamic vesicular trafficking pathways. The membrane-bound and soluble proteins important for these processes are frequent targets of bacterial effectors (58, 85). Indeed, Mtb is known to target and manipulate trafficking pathways through incompletely understood mechanisms (82, 86, 87). To determine whether Mtb proteins can broadly affect the host vesicular trafficking pathways as an indicator of interaction with membranes, we took advantage of a reverse dimerization system (88). In this system, a protein detectable by ELISA is sequestered in the ER by fusion to a conditional aggregation domain (CAD). Addition of a solubilization molecule that disrupts the CAD then frees the fusion protein for trafficking and release into the extracellular space. We used a fusion of human growth hormone (hGH) to the CAD domain of the ligand-reversible cross-linking protein, FKBP F36M. Thus, hGH can be quantified in cell supernatants by ELISA after the addition of the small molecule D/D Solubilizer (Fig. 3A) (88, 89). Another advantage of this system is that it permits determination of the impact of expressed proteins on vesicular trafficking events independent of the bacterial effect on host innate immune responses. We transfected HeLa cells expressing hGH-CAD with each MSP individually, a negative-control protein (GFP), or an enterohemorrhagic Escherichia coli effector (EspG) that inhibits vesicular trafficking by promoting the tethering of vesicles to the Golgi apparatus (89, 90). When we treated transfected cells with D/D Solubilizer, we observed increased, decreased, and normal hGH release (Fig. 3B and Table S1). Using a cutoff of normalized hGH release below 0.25 or above 1.75, we identified 18 proteins that decreased hGH release and 11 proteins that increased hGH release compared to the GFP control (Fig. 3C). We next compared the MSPs that altered host vesicular trafficking to those that bound eukaryotic membranes and identified five proteins with overlapping activities: Rv0594, Rv1646, Rv1810, Rv1980c, and Rv2295 (Fig. 3D). During expression in HeLa cells, all but one protein localized to the ER, and all five proteins reduced hGH release (Fig. 3E).

### Mpt64 N terminus is important for ER localization and inhibition of vesicular trafficking.

We focused on the protein Rv1980c, also known as Mpt64, as it is a secreted protein that is highly antigenic during human tuberculosis infection (91, 92). Furthermore, *mpt64* is a component of the region of difference 2 (RD2) locus, one of the genomic regions deleted during attenuation of the M. bovis BCG vaccine strain (93). Loss of RD2 from Mtb attenuates its virulence, and complementation with a three-gene cluster that includes *mpt64* can partially restore virulence (94).

**FIG 4:**
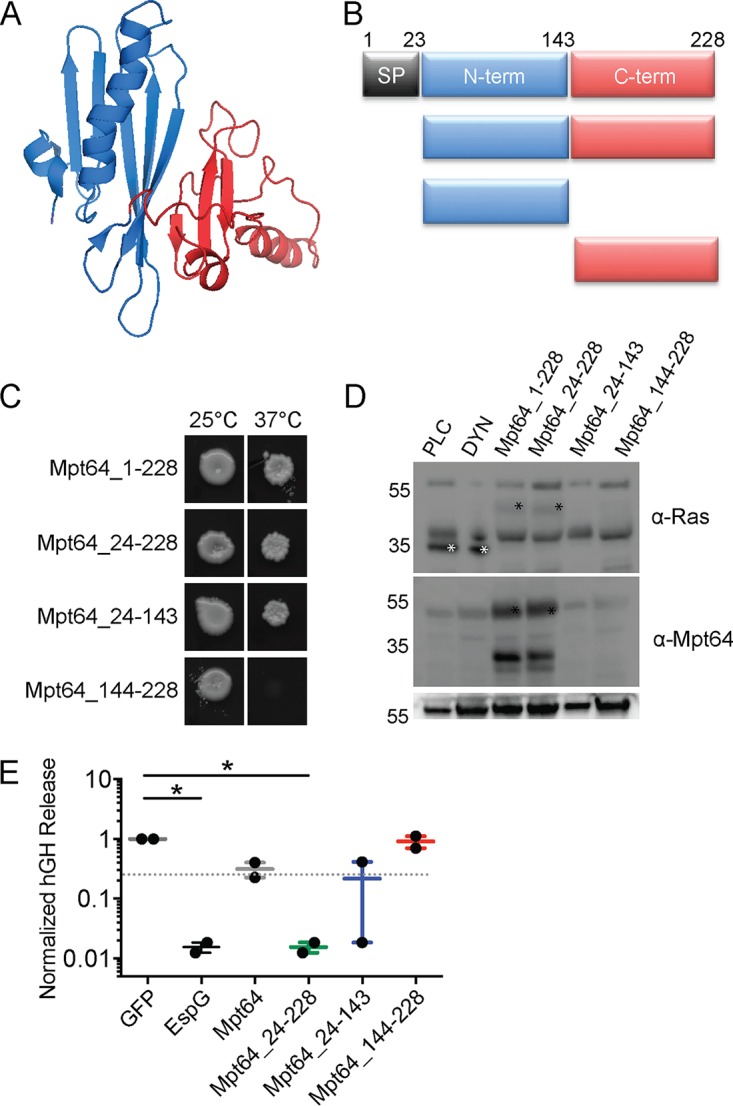
hGH release inhibition is dependent on membrane localization of Mpt64. (A) Solution structure of Mpt64 (PDB accession no. 2HHI). (B) Schematic of Mpt64 truncations, colored to match the solution structure in panel A. SP, signal peptide; N-term, N terminus; C-term, C terminus. (C) Full-length Mpt64 or protein truncations expressed in the Ras rescue assay. The results from one of two independent experiments are shown. (D) Western blot of lysates from cdc25^ts^ yeast expressing Ras^mut^ fusion proteins to control proteins phospholipase C (PLC) and dynamin (DYN) or Mpt64 or Mpt64 truncations. Blots were probed with rabbit anti-Ras, anti-Mpt64, or anti-G6PDH antibodies. Control protein bands are marked by a white asterisk, and Mpt64 truncations are marked by a black asterisk. The image is representative of three experiments. (E) ELISA results of hGH in supernatants of cells coexpressing full-length Mpt64, Mpt64 truncations, or controls. Data are from one representative experiment plotted as box and whiskers from two (Mpt64_24-143 and Mpt64_144-228) or four (Mpt64 and Mpt64_24-228) total experiments. Values that are significantly different (*P* = 0.02) by ANOVA with Dunnett’s multiple-comparison test are indicated by a bar and asterisk.

Mpt64 is a 25-kDa protein with a predicted signal peptidase 1 cleavage site between amino acids 23 and 24, such that the mature, secreted form of the protein starts at amino acid 24 (61, 62, 95). While the solution structure of Mpt64 was previously solved (96), the structure does not align to a known catalytic domain but does contain a domain of unknown function (DUF3298). This domain is also present in the lysozyme-binding anti-sigma factor RsiV (97). Despite structural homology between Mpt64 and RsiV (see Fig. S1 in the supplemental material) (98), there is little primary sequence homology. To determine whether Mpt64 binds lysoszyme, we purified recombinant Mpt64 from E. coli and tested binding to human or hen egg white lysozyme in an *in vitro* pulldown assay (97). Using this assay, we were unable to demonstrate lysozyme binding by Mpt64 (Fig. S1). We next used the solution structure to guide truncation analysis of Mpt64 in order to identify the membrane-binding sequences of Mpt64 (Fig. 4A and B). S. cerevisiae *cdc25^ts^* expressing a fusion of Ras^mut^ with either full-length Mpt64, mature Mpt64 lacking its predicted signal peptide, or the N-terminal half of Mpt64 also lacking the signal peptide (Mpt64_24-143) were able to grow at 37°C, whereas S. cerevisiae *cdc25^ts^* expressing Ras^mut^ fused to the C-terminal half of Mpt64 (Mpt64_144-228) could not (Fig. 4C). We detected expression of Ras^mut^ fusions of full-length Mpt64 and mature Mpt64 by Western blotting. In contrast, we could not detect Ras^mut^ fusions of Mpt64_24-143 or Mpt64_144-228 despite the fact that the Mpt64_24-143 fusion rescued yeast growth, suggesting that expression of Mpt64_24-143 below the limit of detection by Western blotting was still sufficient to rescue yeast growth (Fig. 4D). However, we could not determine whether the C-terminal domain plays a role in membrane binding because we were unable to demonstrate stable fusion protein expression in yeast.

10.1128/mSphere.00354-19.1 FIG S1 Recombinant Mpt64 does not interact with lysozyme. (A) Sequence and secondary structure alignment of Mpt64 and Bacillus subtilis RsiV (template) performed by Phyre^2^ software (http://www.sbg.bio.ic.ac.uk/phyre2/). The Mpt64 DUF3298 is indicated by a red line above the Mpt64 amino acid numbering. (B) Mpt64 (M) or an unrelated protein Cor (C) were immobilized on cobalt beads and hen egg white (HEW) or human lysozyme (HuLYZ) was incubated with these proteins or beads alone (B) for five minutes. After the proteins were washed, they were eluted with 300 mM imidazole. Download FIG S1, TIF file, 13.3 MB. Copyright © 2019 Stamm et al. 2019 Stamm et al. This content is distributed under the terms of the Creative Commons Attribution 4.0 International license.

Finally, we sought to determine whether the N-terminal portion of Mpt64 was also sufficient to inhibit hGH release using the hGH-CAD assay. We cotransfected HeLa cells with plasmids for expression of hGH-CAD and Mpt64 truncation alleles and determined their ability to inhibit hGH release in the presence of drug. Similar to the Ras rescue assay, full-length Mpt64, mature Mpt64, and Mpt64_24-143 inhibited hGH release compared to the GFP control. In contrast, cotransfection of Mpt64_144-228 with hGH-CAD had no effect on its release (Fig. 4E). These data suggest that the ability of Mpt64 to bind membranes and to inhibit host vesicular trafficking *in vitro* is dependent on the N terminus of the protein.

### Mpt64 ER localization depends on its N terminus.

**FIG 5:**
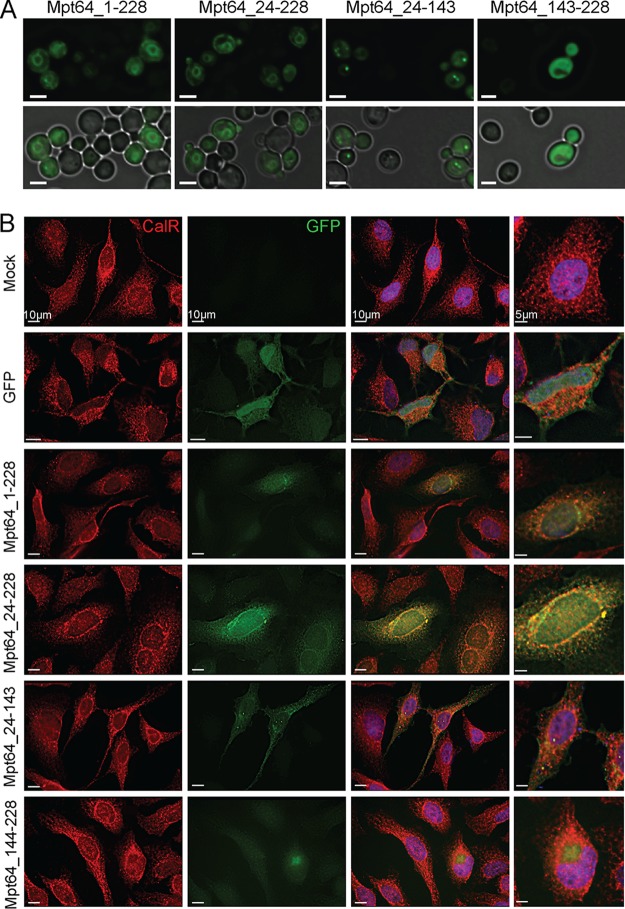
Mpt64 localizes to the endoplasmic reticulum during heterologous expression in yeast and HeLa cells. (A) Immunofluorescence (top panels) and bright-field overlay (bottom panels) images of S. cerevisiae transformed with GFP fusion proteins to Mpt64 truncations. Images are representative of three independent experiments. Bars, 3 μm. (B) HeLa cells transfected overnight with GFP alone (green) or GFP-Mpt64 fusion proteins (green) and stained for ER localization with anti-calreticulin antibody (red). Nuclei are stained with DAPI (blue). Images are representative of two independent experiments. Ten fields of about five cells each were observed for colocalization. Bars, 10 μm.

As full-length Mpt64 localized to the ER in yeast and HeLa cells (Fig. 1 and 3), we next tested the impact of Mpt64 truncations on ER localization. We first determined the phenotypic localization of Mpt64 truncations expressed as GFP fusions in yeast using fluorescence microscopy. Mpt64_1-228 and Mpt64_24-228 localized in a ring indicative of the ER (99, 100) (Fig. 5A). In contrast, Mpt64_144-228, which did not rescue yeast growth in the Ras rescue assay (Fig. 4C), was diffuse throughout the yeast cell (Fig. 5A). Interestingly, Mpt64_24-143 localized to bright puncta within the cells (Fig. 5A). To confirm the N-terminal dependence of Mpt64 localization, we transfected HeLa cells with GFP fusions to each Mpt64 truncation or GFP alone and assayed for colocalization with calreticulin through immunofluorescence microscopy. While full-length Mpt64, mature Mp64, and Mpt64_24-143 colocalized with calreticulin, GFP did not (Fig. 5B). Mpt64_144-228 localized to a bright aggregate that did not colocalize with calreticulin, suggesting that the C terminus of Mpt64 is misfolded and/or unstable when expressed on its own. These results demonstrate that Mpt64 localizes to the ER during exogenous expression in both yeast and mammalian cells and that the N-terminal 143 amino acids are sufficient to mediate subcellular localization of Mpt64 to the ER.

### Mpt64 interacts with phosphatidylinositol phosphates *in vitro*.

**FIG 6:**
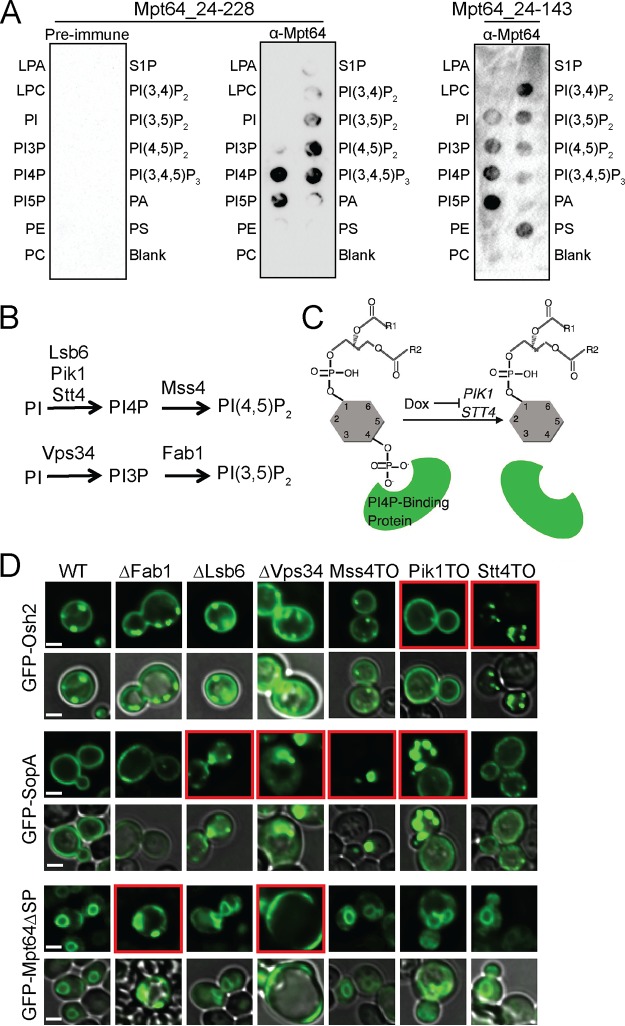
Mpt64 binds phosphatidylinositol phosphates to mediate its ER localization. (A) PIP strips membranes incubated with recombinant Mpt64_24-228 or Mpt64_24-143. Binding of Mpt64 to lipids was detected by incubation with anti-Mpt64 (α-Mpt64) or preimmune serum. Abbreviations indicate specific lipids as follows: LPA, lysophosphatidic acid; LPC, lysophosphatidylcholine; PI, phosphatidylinositol; PI3P, phosphatidylinositol-3-phosphate; PI4P, phosphatidylinositol-4-phosphate; PI5P, phosphatidylinositol-5-phosphate; PE, phosphatidylethanolamine; PC, phosphatidylcholine; S1P, sphingosine 1-phosphate; PI(3,4)P_2_, phosphatidylinositol-3,4-bisphosphate; PI(3,5)P_2_, phosphatidylinositol-3,5-bisphosphate; PI(4,5)P_2_, phosphatidylinositol-4,5-bisphosphate; PI(3,4,5)P_3_, phosphatidylinositol-3,4,5-trisphosphate; PA, phosphatidic acid; PS, phosphatidylserine. Images are representative of two (Mpt64_24-143) or four (Mpt64_24-228) independent experiments. (B) PIP synthesis is regulated by six PI kinases in yeast by the indicated pathways. (C) Design of the PI kinase experiment. Representative results for Osh2, a PI4P-binding protein, are shown. Doxycycline (Dox) repression of a PI kinase (PIK1 and STT4 are shown) depletes the PIP, causing loss of localization of proteins that have a membrane localization governed by PI4P (i.e., Osh2). (D) Localization of GFP-Osh2 (a known PI4P-binding protein), GFP-SopA (a promiscuous PIP-binding protein), or GFP-Mpt64 in wild-type S. cerevisiae cells and the six PI kinase yeast strains. Images are representative of two (FAB1, LSB6, and VPS34) or three (MSS4, PIK1, and STT4) independent experiments.

To test whether Mpt64 could interact with lipids directly, we expressed and purified recombinant Mpt64 and Mpt64 variants from E. coli (Fig. S2) and tested their ability to bind unique lipid species *in vitro* using membranes spotted with lipids. Recombinant Mpt64_24-228 bound phosphatidylinositol 4-phosphate (PI4P), PI5P, phosphatidylinositol 3,5-bisphosphate [PI(3,5)P_2_], PI(4,5)P_2_, and phosphatidylinositol (3,4,5)-trisphosphate [PI(3,4,5)P_3_] on PIP strips membranes (Fig. 6A). Similarly, recombinant Mpt64_24-143, the N-terminal portion of the protein, also bound PI4P and PI5P with additional binding to PI3P, PI(3,4)P_2_, and phosphatidylserine (Fig. 6A). However, interaction with PI(4,5)P_2_ and PI(3,4,5)P_3_ was weak, suggesting that the C-terminal region of Mpt64 modifies its interactions with host phospholipids. We were unable to test PIP binding by Mpt64_144-228 because its expression in E. coli was weak and the protein was insoluble after purification using similar conditions for Mpt64_24-228 and Mpt64_24-143.

10.1128/mSphere.00354-19.2 FIG S2 Expression and purification of recombinant Mpt64 truncations. (A) Detection of recombinant Mpt64 protein expression by PAGE, followed by Coomassie brilliant blue stain. (B) Detection of recombinant Mpt64 protein expression by Western blotting. Mpt64_24-228 (ΔSP), Mpt64_24-143 (N terminus [NT]), and Mpt64_144-228 (C terminus [CT]) were detected by anti-Mpt64. Download FIG S2, TIF file, 13.7 MB. Copyright © 2019 Stamm et al. 2019 Stamm et al. This content is distributed under the terms of the Creative Commons Attribution 4.0 International license.

### Mpt64 ER localization in yeast is dependent on PI3P and PI(3,5)P_2_.

On the basis of the results from the PIP strips (Fig. 6A) and to further characterize the lipid binding of Mpt64 *in vivo*, we took advantage of yeast strains mutated in phosphatidylinositide (PI) kinases that either lack or have reduced levels of specific phosphatidylinositol phosphates (PIPs) (Fig. 6B). Because PIPs are geographically restricted within cells and their position-specific phosphorylation patterns can function as organelle-specific markers to recruit proteins to areas with unique membrane constituents (57, 101, 102), inactivation of yeast PI kinases causes mislocalization of bacterial effectors that need such PIP interactions for appropriate membrane targeting (57) (Fig. 6C). We inhibited expression of each yeast PI kinase gene either by isogenic knockout of the nonessential PI kinase gene (VPS34, FAB1, and LSB6) or by doxycycline (Dox)-mediated repression of TetO_7_-promoter alleles of essential PI kinase genes (PIK1, STT4, and MSS4). We optimized repression conditions for the TetO_7_-promoter alleles by monitoring the distribution of the PI4P-specific binding protein Osh2, which shuttles between the plasma membrane and Golgi apparatus in a PI4P-dependent manner (Fig. 6D) (57, 103, 104). Thus, Dox-mediated loss of PIK1 and STT4 expression led to Osh2 redistribution to the plasma membrane (PM) or Golgi apparatus, respectively, as previously reported (57). For a further control for the isogenic deletion strains, we observed that deletion of LSB6 and VPS34 along with Dox-mediated loss of MSS4 and PIK1 caused relocalization of the *S.* Typhimurium effector SopA from the PM to internal puncta, consistent with its affinity for several PIP isoforms (57). Using this assay, Mpt64 relocalized from the ER to the PM and internal puncta in the absence of VPS34 and FAB1 (Fig. 6D). As these strains lack PI3P and PI(3,5)P_2_ and because the VPS34 strain lacks both PIPs while the FAB1 strain lacks only PI(3,5)P_2_, we conclude that recruitment of Mpt64 to the ER in yeast is likely dependent on PI(3,5)P_2_. Though the PIP strips (Fig. 6A) showed strong binding to PI4P, we propose that the failure to relocalize in yeast lacking various PI kinases that produce PI4P (i.e., LSB6, PIK1, and STT4) is due to their redundancy. Taken together, the data indicate that Mpt64 interacts with PIPs *in vitro* and *in vivo* to facilitate its localization to the ER.

### Secreted Mpt64 localizes to the ER during infection.

**FIG 7:**
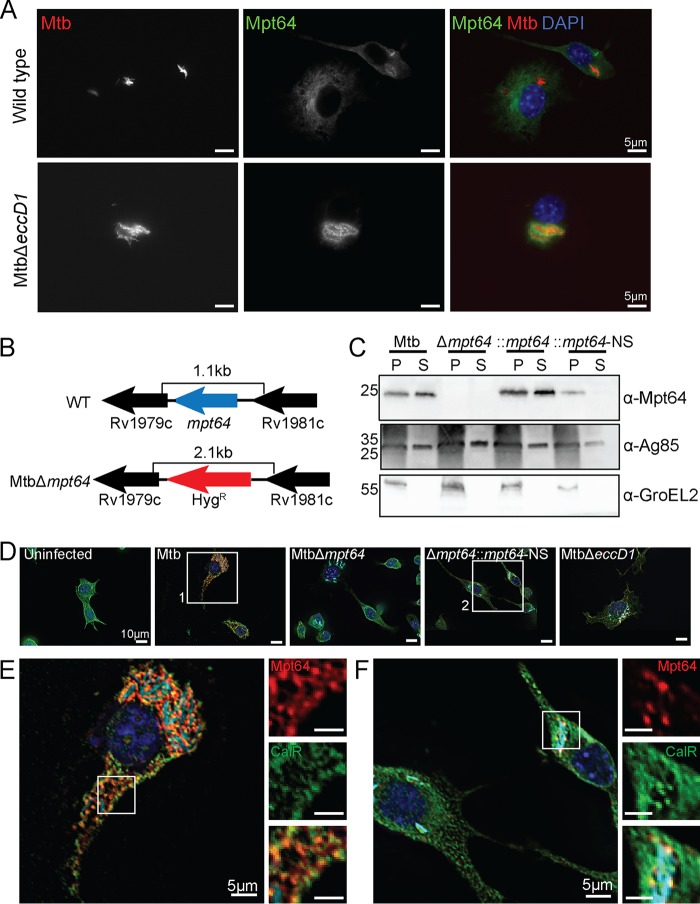
Mpt64 ER localization is ESX1 dependent during M. tuberculosis infection of macrophages. (A) RAW267.4 murine macrophages were infected with mCherry-expressing (red) wild type (top panels) or MtbΔ*eccD1* (bottom panels) for 4 h at an MOI of 20:1. Cells were fixed and stained for Mpt64 (green) and nuclei (blue). Bars, 5 μm. (B) Schematic detailing in-frame deletion of *mpt64* by insertion of a hygromycin resistance gene. (C) Representative Western blot from one of three experiments detecting expression of Mpt64, Ag85, and GroEL2 in either the lysate of the cell pellet (P) or culture supernatant (S) of four Mtb strains. (D) RAW267.4 macrophages were infected with the indicated strains of mCherry-expressing (cyan) Mtb for 4 h at an MOI of 20:1. Cells were fixed and stained for Mpt64 (red), calreticulin (green), and nuclei (blue). Bars, 10 μm. (E) Enlarged image from box 1 in panel D of an Mtb-infected macrophage stained for Mpt64, calreticulin, and DAPI. The insets show an area of Mpt64-calreticulin colocalization. Bars, 5 μm. (F) Enlarged image from box 2 in panel D of macrophages infected with MtbΔ*mpt64*::*mpt64*-NS and stained for Mpt64, calreticulin, and DAPI. The insets show Mpt64 localization in relation to bacteria. Bars, 5 μm. Images in panels A and D to F are representative of one of three experiments. Ten fields of about five cells each were observed for colocalization.

Although we observed Mpt64 localization to the ER in yeast (Fig. 1E and Fig. 5A) and HeLa cells (Fig. 5B), we wanted to determine whether endogenous, untagged Mpt64 localizes to the ER during an Mtb infection of macrophages. To that end, we infected mouse RAW267.4 macrophages with mCherry-labeled Mtb at a multiplicity of infection (MOI) of 20:1 and fixed cells at various time points after infection. We then used a rabbit polyclonal antibody developed against recombinant, mature Mpt64 protein to track Mpt64 secretion from Mtb into macrophages using immunofluorescence microscopy. Of note, this antibody was generated without complete Freund’s adjuvant in order to avoid any cross-reactivity against Mtb antigens generated by the use of this adjuvant. As little as 4 h after infection, endogenous Mpt64 was detected in the cytoplasm host cells in a lacy pattern indicative of ER localization (Fig. 7A, top panels). When we infected macrophages with MtbΔ*eccD1*, a strain that is deficient in ESX-1 secretion (105, 106) and does not result in communication between the phagosome and cytoplasm (27, 45, 46, 107–109), Mpt64 appeared to be secreted but trapped adjacent to the bacteria (Fig. 7A, bottom panels), suggesting that it could not escape the phagosome. Importantly, Mpt64 was detected in the culture filtrate prepared from MtbΔ*eccD1* (Fig. S3). Thus, although Mpt64 is likely secreted from Mtb by the canonical Sec-dependent pathway, its access to the macrophage cytoplasm and other targets in the cell was dependent on the type VII secretion system.

10.1128/mSphere.00354-19.3 FIG S3 Construction and phenotypic analysis of MtbΔ*mpt64*. (A) Western blot detecting expression of Mpt64, Ag85, and GroEL2 in either the lysate of the cell pellet (P) or culture supernatant (S) of wild-type Mtb or MtbΔ*eccD1*. (B) Detection of hygromycin resistance cassette insertion in place of *mpt64*. Genomic DNA from wild-type Mtb or MtbΔ*mpt64* was amplified by polymerase chain reaction, and products were analyzed by agarose gel electrophoresis. (C) Growth of Mtb, MtbΔ*mpt64*, MtbΔ*mpt64*::*mpt64*, and MtbΔ*mpt64*::*mpt64*-NS in 7H9 measured by optical density at 600 nm. (D) PDIM standard or apolar lipid extracts from Mtb or MtbΔ*mpt64* were analyzed on an AbSciex TripleTOF 5600/5600+ mass spectrometer. Download FIG S3, TIF file, 9.9 MB. Copyright © 2019 Stamm et al. 2019 Stamm et al. This content is distributed under the terms of the Creative Commons Attribution 4.0 International license.

In order to better understand the role of Mpt64 in Mtb virulence, we used mycobacteriophage (110–112) to introduce the hygromycin resistance cassette into the *mpt64* gene to create an in-frame deletion (Fig. 7B). We confirmed disruption of *mpt64* by PCR (Fig. S2) and loss of Mpt64 by the absence of protein on Western blots (Fig. 7C). We then complemented MtbΔ*mpt64* with either full-length *mpt64* (MtbΔ*mpt64*::*mpt64*) or Mpt64 lacking its signal peptide (MtbΔ*mpt64*::*mpt64*-NS) under the control of the constitutive mycobacterial strong promoter (113). Both complemented strains expressed Mpt64, but only full-length Mpt64 (MtbΔ*mpt64*::*mpt64*) could be detected in the supernatant of cultures, confirming that deletion of the signal peptide inhibits Mpt64 secretion from Mtb (Fig. 7C). Furthermore, the MtbΔ*mpt64*::*mpt64* strain had modestly higher expression of Mpt64 compared to wild-type Mtb by Western blotting, consistent with our use of a strong constitutive promoter for complementation. All four strains grew equally under axenic growth conditions (Fig. S3), and we confirmed that both Mtb and MtbΔ*mpt64* produced phthiocerol dimycocerosate (PDIM) by mass spectrometry (Fig. S3).

To test whether secreted Mpt64 localizes to the ER during infection, we assessed its colocalization with calreticulin in RAW267.4 cells using confocal immunofluorescence microscopy. When we infected RAW267.4 macrophages, the Mpt64 signal in Mtb-infected macrophages colocalized with calreticulin, confirming the subcellular localization of Mpt64 secreted during infection (Fig. 7D and E and Fig. S3). However, this colocalization was lost in cells infected with MtbΔ*mpt64*::*mpt64*-NS bacteria (Fig. 7D and F and Fig. S4). For a control for antibody specificity, no Mpt64 was detected in macrophages infected with MtbΔ*mpt64* mutant bacteria (Fig. 7D and Fig. S4). From these data, we can confirm that the signal peptide of Mpt64 is sufficient for the protein’s secretion *in vivo* and is required (with concerted action of the type VII secretion system) for Mpt64 to localize to the ER during infection.

10.1128/mSphere.00354-19.4 FIG S4 Secreted Mpt64 colocalizes with calreticulin in murine macrophages. RAW267.4 murine macrophages were infected with the indicated strains of mCherry expressing Mtb (cyan) for four hours and subsequently stained for Mpt64 (red) and calreticulin (green). Nuclei are stained in blue. Scale bars are 10 μm. Download FIG S4, TIF file, 11.1 MB. Copyright © 2019 Stamm et al. 2019 Stamm et al. This content is distributed under the terms of the Creative Commons Attribution 4.0 International license.

### Mpt64 inhibits the unfolded protein response.

**FIG 8:**
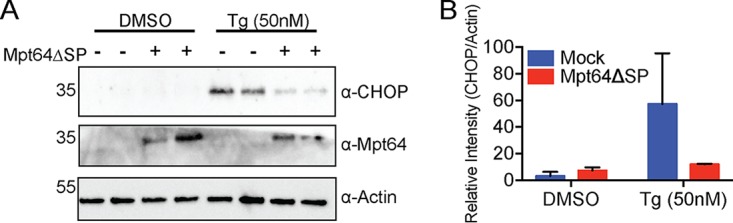
Mpt64 inhibits the unfolded protein response. (A) RAW267.4 cells stably transduced with empty lentivirus or lentivirus expressing Mpt64ΔSP under the control of the CMV promoter were treated with DMSO or thapsigargin (Tg) (50 nM) for 4 h, and CHOP protein accumulation was detected by Western blotting. (B) Quantitative densitometry analysis of the Western blot in panel A. The results of one experiment representative of >3 experiments are shown.

ER stress and the UPR have recently been associated with Mtb infection and pathogenesis (114–116). Because we found that Mpt64 localizes to the ER during infection, we hypothesized that it might regulate the UPR. To test whether Mpt64 was sufficient on its own to impact the UPR, we stably transduced murine RAW267.4 cells with empty lentivirus or a lentivirus with Mpt64 under the control of a cytomegalovirus (CMV) promoter and induced the UPR by treating with thapsigargin, a known UPR activator (117). In the presence of thapsigargin, we detected robust accumulation of the UPR-activated transcription factor CCAAT enhancer-binding protein homologous protein (CHOP), a protein whose expression is low under nonstressed conditions but high in the setting of ER stress (117) (Fig. 8A). Expression of Mpt64 resulted in a 75% reduction in CHOP compared to control cells, indicating that Mpt64 alone could inhibit the UPR (Fig. 8B).

### Mpt64 contributes to early Mtb growth after aerosol infection of mice.

**FIG 9:**
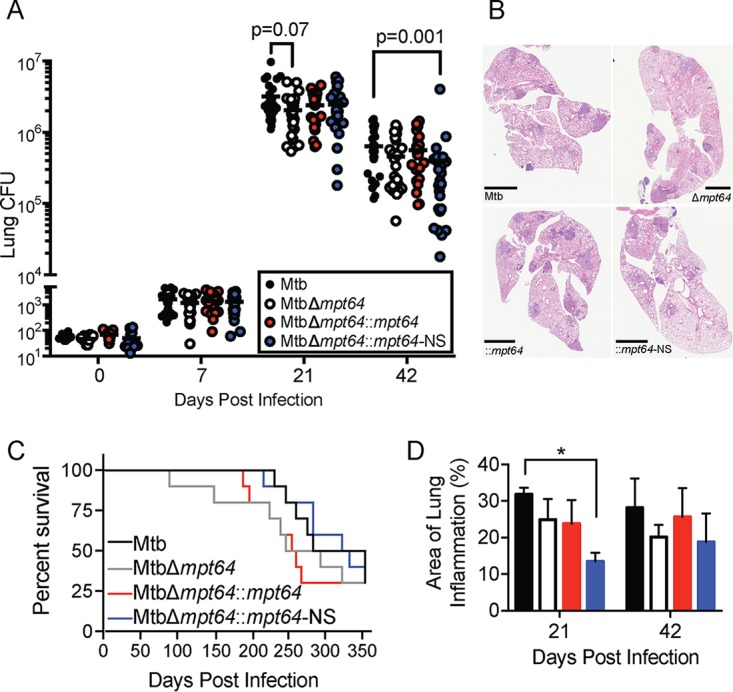
Mpt64 contributes to early Mtb growth after aerosol infection of mice. (A) Bacterial burden in lungs of mice 0, 7, 21, and 42 days after aerosol infection with the indicated Mtb strains. Results are combined from three independent experiments with 25 mice total per group. The horizontal bar indicates the geometric mean. *P* values were determined by the nonparametric Kruskal-Wallis test. (B) Representative images of H&E-stained lungs at 42 days postinfection with the indicated strains of Mtb. Bars, 2 mm. (C) Ten mice per group were monitored for survival. There were no significant differences in survival rates between groups by Kaplan-Meier analysis. (D) Quantitation of lung inflammation of mice infected with the indicated Mtb strains. Measurement was determined using ImageJ software (NIH). Bars are colored as in panel A. Values are means plus standard errors of the means (SEM) (error bars) for three animals per group. Values that are significantly different (*P* < 0.02) by Kruskal-Wallis test are indicated by a bar and asterisk.

Because *mpt64* is part of the Mtb RD2 locus that partially accounts for the attenuation of Mtb (94) and our data indicating that Mpt64 may function as a secreted effector that modulates the UPR, we investigated the role of Mpt64 in Mtb virulence in a murine model of infection. We infected BALB/c mice via aerosol with a low bacterial inoculum (∼50 to 100 CFU Mtb) and collected lungs at various time points to determine CFU and histopathology. We compared the infections of four strains: wild-type Mtb, MtbΔ*mpt64*, MtbΔ*mpt64*::*mpt64*, and MtbΔ*mpt64*::*mpt64*-NS. While all mice received equal numbers of bacteria of the four strains at day 0, there were one-third fewer Mtb isolated from the lungs of mice infected with the MtbΔ*mpt64* mutant compared to mice infected with the wild type at 21 days (mean CFU of 2.7 × 10^6^ for wild-type Mtb versus 1.7 × 10^6^ for MtbΔ*mpt64*; *P* = 0.07) and 42 days (mean CFU of 5.0 × 10^5^ for wild-type Mtb versus 3.4 × 10^5^ for MtbΔ*mpt64*) postinfection. Though these effect sizes were modest and consistent for both time points, they did not meet statistical significance at an alpha of *P < *0.05. By 42 days postinfection, we observed statistically significant decreases in the CFU isolated from lungs of mice infected with the MtbΔ*mpt64*::*mpt64*-NS strain (mean CFU of 5.0 × 10^5^ for wild-type Mtb versus 1.8 × 10^5^ for MtbΔ*mpt64*::*mpt64*-NS; *P* = 0.001) (Fig. 9A). At these time points, we also observed a reduction in the area of inflammation in hematoxylin-and-eosin (H&E)-stained lungs of mice infected with MtbΔ*mpt64*::*mpt64*-NS compared to mice infected with the wild type (Fig. 9B and D). Despite modest reductions in CFU in the lungs of mice infected with mutant bacteria, there was no impact on mouse survival (Fig. 9C).

### Mpt64 localized to the ER in primary human macrophages but is dispensable for Mtb survival.

**FIG 10:**
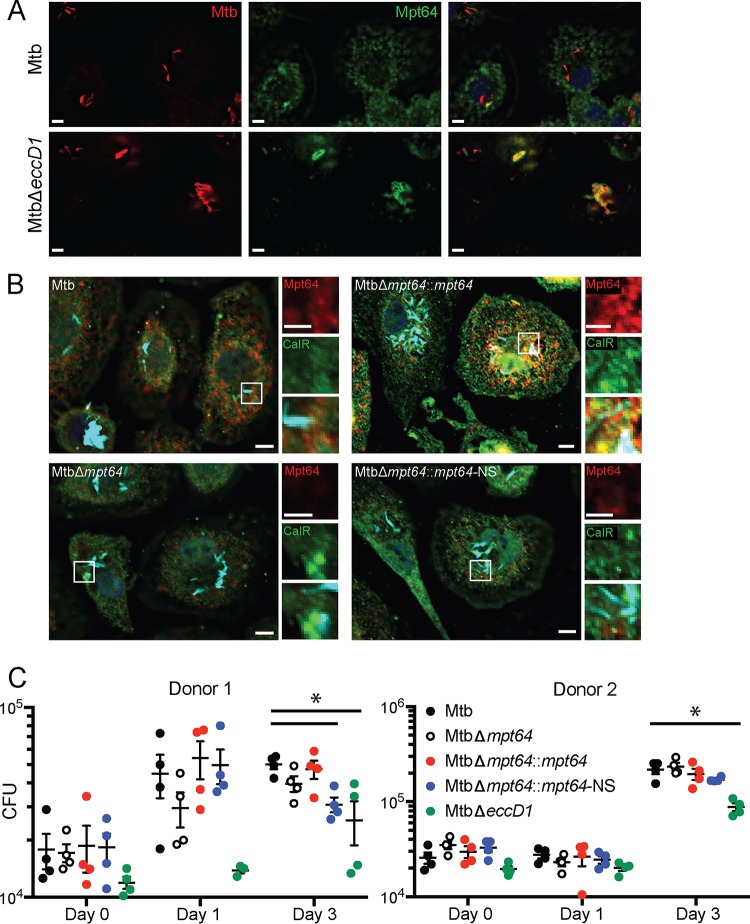
Mpt64 localization and impact on survival in primary human macrophages. (A) Primary human monocyte-derived macrophages were infected with mCherry-expressing (red) Mtb or MtbΔ*eccD1* for 4 h at an MOI of 10:1. Cells were fixed and stained for Mpt64 (green). Bars, 5 μm. (B) Primary human monocyte-derived macrophages were infected with the indicated strains of mCherry-expressing Mtb for 4 h at an MOI of 10:1. Cells were fixed and stained for Mpt64 (red) and calreticulin (green). Nuclei are stained blue. Bars, 5 μm. The images in panels A and B are representative of three independent experiments. Ten fields of about five cells each were observed for colocalization. (C) CFU recovered from primary human macrophages from two independent donors infected with Mtb, MtbΔ*mpt64*, MtbΔ*mpt64*::*mpt64*, MtbΔ*mpt64*::*mpt64*-NS, or MtbΔ*eccD1* at the indicated time points. Each symbol represents the value for a biological replicate at each time point per strain, and the bars indicate the mean with standard error. The results for two donors of four donors are shown. *, *P* = 0.02 by Kruskal-Wallis test with Dunn’s correction for multiple comparisons.

Next, we assessed whether the localization of Mpt64 in human cells is similar to that in murine macrophages. To that end, we infected primary human monocyte-derived macrophages with mCherry-expressing wild-type (WT) Mtb or MtbΔ*eccD1* and stained for Mpt64. Consistent with our data in RAW267.4 cells (Fig. 7A), Mpt64 localization to extraphagosomal sites in primary human macrophages was dependent on the type VII secretion system (Fig. 10A). We then infected primary human macrophages with WT Mtb, MtbΔ*mpt64*, MtbΔ*mpt64*::*mpt64*, or MtbΔ*mpt64*::*mpt64*-NS and determined the colocalization of Mpt64 with calreticulin by fluorescence microscopy (Fig. 10B and Fig. S5). At 4 h postinfection (hpi), we detected colocalization of Mpt64 with calreticulin in cells infected with WT Mtb and MtbΔ*mpt64*::*mpt64* but not in cells infected with MtbΔ*mpt64* or MtbΔ*mpt64*::*mpt64*-NS (Fig. 10B).

10.1128/mSphere.00354-19.5 FIG S5 Secreted Mpt64 colocalizes with calreticulin in human macrophages. Primary human macrophages were infected with the indicated strains of mCherry expressing Mtb (cyan) or left uninfected for four hours prior to fixation and staining for Mpt64 (red) and calreticulin (green). Scale bars are 5 μm. Download FIG S5, TIF file, 8.5 MB. Copyright © 2019 Stamm et al. 2019 Stamm et al. This content is distributed under the terms of the Creative Commons Attribution 4.0 International license.

To better understand the contribution of Mpt64 in the context of human Mtb infection, we determined the growth of wild-type Mtb, MtbΔ*mpt64*, MtbΔ*mpt64*::*mpt64*, MtbΔ*mpt64*::*mpt64*-NS, and MtbΔ*eccD1* as a control for attenuation in primary monocyte-derived human macrophages during acute infection. We recovered CFU from cells directly after infection (day 0) and 1 and 3 days postinfection. We observed significant donor-to-donor variability both in the ability to restrict intracellular Mtb replication (compare the growth of WT Mtb between representative donor 1 and donor 2) and the relative growth of MtbΔ*mpt64*, MtbΔ*mpt64*::*mpt64*, and MtbΔ*mpt64*::*mpt64*-NS in various donors. Thus, while in some donors, the CFU at day 3 postinfection of strains lacking *mpt64* was modestly but not statistically significantly lower compared to WT Mtb (i.e., donor 1), in other donors, there was no impact on the presence or absence of *mpt64* (i.e., donor 2). Thus, in this acute primary human macrophage infection model, the presence of Mpt64 appeared to be dispensable for Mtb survival.

## DISCUSSION

Numerous efforts have been undertaken to identify Mtb secreted proteins, from lipoproteins that are incorporated into the cell wall to virulence factors that reach the extracellular environment such as ESAT-6 (59–61). However, little is known about the function of this “exoproteome” as a whole. Here we took a systematic approach toward characterizing host-dependent interactions of a collated list of putative secreted Mtb proteins. We created a library of 200 putative secreted proteins and then through a series of cell biological screens characterized these MSPs for their ability to bind eukaryotic membranes, their subcellular localization, and their ability to modulate release of a model substrate. In addition, we demonstrate that one secreted protein, Mpt64, localized to the ER during infection of mouse and human macrophages and inhibited the UPR. The cohort of 200 MSPs we generated was large but not necessarily exhaustive (see Table S1 in the supplemental material). For example, the 76 PE/PPE genes we included represent less than half (45%) of the total number of PE/PPE genes in the Mtb genome (118). In addition, a recent technology called exported *in vivo* technology (EXIT) identified 593 Mtb proteins secreted during intravenous infection of mice, including 38 proteins that are significantly enriched only during *in vivo* infection compared to growth on Middlebrook 7H10 agar, suggesting a virulence function for these proteins (67). Of the 200 MSPs we characterized, 51 overlap with those identified by EXIT, and of the 51 overlapping proteins, 25 are membrane associated in our study. This emphasizes that host membranes can be targets of Mtb secreted proteins.

We found 52 Mtb proteins that associated with eukaryotic membranes, representing nearly 25% of the total screened. When the membrane association of type III and type IV effectors from several Gram-negative pathogens was explored, about 30% of effectors screened also associated with eukaryotic membranes (57). While our data are in agreement with this value, pathogens that replicate intracellularly in vacuoles had even higher numbers of membrane-associated effectors (57). This suggests that there may be additional secreted virulence proteins from Mtb that associate with the host membranes than our screen identified. Indeed, while we corroborated previously known membrane-interacting proteins such as the SecA2 secreted PI3P phosphatase SapM (17, 18), the Rac1-binding protein Ndk (119), and the cholesterol-binding Mce4A (120), we failed to identify others such as LipY which hydrolyzes extracellular lipids (121) and the ESX1 substrate ESAT-6, whose ability to directly interact with the phagosomal membrane (122, 123) has recently been questioned (65, 124).

The vast majority of MSPs localized to the ER when expressed in HeLa cells. Whether this localization reflects the importance of modulating ER function for Mtb survival cannot be determined directly, as we did not raise protein-specific antibodies against the proteins like we did for Mpt64 in order to determine the localization of untagged, endogenous, secreted protein during infection. It is possible that some of the observed localizations represent false-positive results, as overexpression of Mtb proteins with signal sequences results in aberrant ER localization in 293T cells (125). In addition, we found some MSPs that rescued yeast growth in the Ras rescue assay but localized to the cytoplasm when expressed as GFP fusions in HeLa cells. These proteins represent either false-positive results from the Ras rescue assay or proteins that do associate with lipids whose abundance is sufficient in yeast cells but not in HeLa cells.

GFP-Mpt64 localized to the ER in both yeast cells and mammalian cells. Additionally, endogenous Mpt64 localized to the ER during Mtb infection of macrophages, suggesting that the observed localization of Mpt64 is not an artifact of heterologous overexpression. Mpt64 did not colocalize with the ER after infection with a Mtb type VII secretion system mutant, underscoring the importance of the phagosome-disrupting properties of the ESX-1 system in establishing communication with the host cell (65, 126). This ESX-1-dependent mechanism of cytoplasmic access is similar to the route taken by the autotransporter-like protein tuberculosis necrotizing toxin (TNT) (127, 128). Thus, our data strengthen the argument that the type VII secretion system facilitates access of non-ESX-1 substrates beyond the phagosome and into the host cell.

Mpt64 bound several PIPs *in vitro* with the most prominent interactions being with the monophosphatidylinositols PI3P, PI4P, and PI5P. Furthermore, the ER localization of Mpt64 in yeast changed when the PIP kinases generating PI3P and PI(3,5)P were deleted, suggesting that its function *in vivo* is connected to its ability to interact with membrane PIPs. While PI3P is mainly detected in endosomes and autophagosomes (129), PI3P has also been identified at specialized ER sites called omegasomes where a dynamic exchange of PI3P-positive vesicles and ER occurs, allowing for assembly of autophagy proteins and expansion of autophagosome membranes, leading to initiation of autophagosome formation (130). PI4P is thought to be enriched in the Golgi apparatus (131), but it also has an established role in mediating protein trafficking from ER exit sites (132, 133). Less is known about PI5P, as its basal level is only about 1% of the basal PI4P level (134). However, PI5P is increased during bacterial infection and other stresses and can be found throughout the cell, including the ER (134) and on lipid droplets that arise from the ER (135). Similarly, PI(3,5)P_2_ is present at low abundance, but its levels are elevated by stress such as hyperosmotic shock in yeast (136). Conserved functions of PI(3,5)P_2_ from yeast to mammals include regulation of autophagy and retrograde trafficking, activation of some ion channels, and cargo sorting into multivesicular bodies (136). Thus, the subcellular localization of Mpt64 at the ER may result from its interaction with the monophosphatidylinositols PI3P, PI4P, and PI5P in addition to PI(3,5)P_2_, thus allowing Mpt64 to interfere with ER to Golgi trafficking and prevent release of the model substrate hGH and also inhibit the UPR. However, the relative contribution of binding to individual PIPs to the activity of Mpt64 remains unknown.

Although an enzymatic activity for Mpt64 could not be deduced from its structure, we were able to demonstrate that the N terminus of Mpt64 was sufficient to mediate membrane binding, interaction with PIPs, and inhibition of vesicular trafficking. Mature Mpt64 was also able to inhibit the UPR in the setting of thapsigargin-induced ER stress in macrophages. Since ER stress has been observed *in vivo* during Mtb infection and ER stress is known to activate autophagy and apoptosis (137), the ability of Mpt64 to downregulate the UPR may allow Mtb to fine-tune the host response in order to provide a long-term replicative niche. Indeed, since both autophagy (138, 139) and apoptosis (140) have critical roles in the outcome of Mtb infection, engagement by Mtb of high-value targets such as the ER, Golgi apparatus, and mitochondria—organelles vital to cellular regulation of autophagy and apoptosis—with its entire secreted effector armament such as the membrane-binding MSPs identified here likely allows Mtb to tightly control the host response to facilitate successful infection.

Disruption of the RD2 locus in Mtb H37Rv leads to decreased bacterial burdens in the lungs and spleen of aerosol-infected mice at 3 weeks after infection (94). As *mpt64* is within the RD2 locus, we hypothesized that the single, in-frame deletion of *mpt64* might explain the attenuation phenotype of the RD2 mutant. Though we did not perform a head-to-head comparison of an MtbΔRD2 strain versus our MtbΔ*mpt64* strain on the Erdman genetic background, we did observe modestly decreased bacterial burdens of MtbΔ*mpt64* compared to WT Mtb in the lungs of mice at 3 weeks postinfection. Other genes located in the RD2 locus that were not complemented in the RD2 survival study (94) such as pe_pgrs35 (Rv1983) and cfp21 (Rv1984) may also contribute to the virulence defect observed in RD2 deletions. Furthermore, it is possible that one or more of the other Mtb secreted proteins we identified, including the 27 proteins that also localized to the ER, are able to perform a redundant function to that of Mpt64 during an animal infection. In a similar vein, whereas L. pneumophila encodes more than 300 effectors, individual L. pneumophila effector deletion mutants are not defective for growth in cells or mice (141, 142). Indeed, a L. pneumophila strain in which 11 effectors that function to inhibit protein translation are deleted is still able to inhibit host protein translation, though a mutant in the type IV secretion machine itself cannot (143), indicating remarkable redundancy in effector activity. Thus, future work disrupting multiple Mtb MSPs simultaneously will help address this issue.

When we infected mice with MtbΔ*mpt64*::*mpt64*-NS, a strain of Mtb that still expresses Mpt64 but cannot secrete it into the host cell, we recovered fewer CFU compared to WT from the lungs of mice infected with MtbΔ*mpt64*::*mpt64*-NS. We hypothesize that this strain suffers from two detrimental consequences. First, blocking Mpt64 secretion prevents it from exerting its function in the host. Second, nonsecreted Mpt64 can still be cross-presented to the adaptive immune system (144), thus leading to a cell-mediated immune response against Mpt64. This observation is consistent with data that both human patients with active tuberculosis and their PPD-positive contacts have T-cell responses to Mpt64 (145) and T-cell-reactive Mpt64 epitopes have been mapped (146). Furthermore, Mpt64 staining is observed in granulomas of infected individuals (147, 148). Thus, Mpt64 is highly immunogenic during human infection with Mtb and suggests an evolutionary trade-off between the effector function of Mpt64 and its antigenicity. When we explored the importance of Mpt64 in human disease, we observed that Mpt64 secreted from wild-type bacteria localized to the ER of infected human monocyte-derived macrophages, though a *mpt64* mutant did not have a consistent defect in survival within macrophages. Future work on other mycobacterial secreted, ER-binding proteins may ultimately reveal functional redundancy with Mpt64 important for the virulence of Mtb.

## MATERIALS AND METHODS

### Bacterial strains and growth conditions.

M. tuberculosis Erdman and mutants were grown in Middlebrook 7H9 broth or on Middlebrook 7H11 agar (Difco) supplemented with 10% oleic acid-albumin-dextrose-catalase (OADC) (Remel). Liquid medium was also supplemented with 0.05% Tween 80.

### Yeast strains and assays.

The Saccharomyces cerevisiae strain INVSc1 (Invitrogen) was grown at 30°C in histidine dropout medium (synthetic defined [SD] base dropping out histidine [SD/−HIS]) or agar plates (Clontech). The construction of the *cdc25^ts^* strain was previously described (80). The *cdc25^ts^* strain was grown at 25°C in leucine dropout medium or agar plates (SD/−LEU) (Clontech). Yeast knockout strains ΔFAB1, ΔLSB6, and ΔVPS34 are on the S. cerevisiae BY4741 background and were grown in YPD (Difco) prior to transformation. The tetracycline off strains (MSS4, PIK1, and STT4; Thermo Fisher Scientific) are on the genetic background of R1158 and were grown in YPD supplemented with 300 μg/ml G418 (catalog no. 345810; Millipore) prior to transformation.

Yeast strains were transformed using a lithium acetate (LiAc) protocol. The yeast strains were grown to high density overnight at the appropriate temperature with shaking. The cultures were diluted to an optical density at 600 nm (OD_600_) of 0.2 in 50 ml YPD and allowed to reach mid-log phase. Cells were washed, resuspended in 0.1 M LiAc, and incubated 10 min at room temperature. The sample DNA was mixed with an equal volume of preboiled Yeastmaker Carrier DNA (Clontech). To the DNA was added 100 μl yeast and 500 μl of a solution of LiAc plus PEG (40% [wt/vol] PEG, 0.1 M LiAc). This solution was incubated 30 min at 25°C (*cdc25^ts^*) or 30°C with agitation every 10 min. DMSO was added, and the cells were heat shocked at 42°C for 15 min. The cells were pelleted, washed in TE (10 mM Tris [pH 7.4], 1 mM EDTA) and resuspended in 500 μl TE. The transformed cells were plated on selective agar plates and incubated at the appropriate temperature for 2 to 4 days.

To perform the Ras rescue assay, three or four fresh colonies were combined in 30 μl SD/−LEU, and 3 μl was spotted onto duplicate plates that were subsequently incubated at either 25°C or at 37°C for 2 days.

INVSc1, deletion mutants and tetracycline off yeast were transformed with a galactose-inducible vector (p413GALGFP) containing GFP-Mtb fusion proteins and selected on SD/−HIS. To induce GFP fusion protein expression in wild-type (WT) and deletion mutant strains, yeast cells were inoculated in 5 ml galactose/raffinose (Gal/Raf) base lacking histidine (Clontech) and allowed to grow for 16 to 20 h at 30°C with shaking. Cultures were pelleted, resuspended in 3 ml Gal/Raf/−HIS and incubated for another 4 h at 30°C with shaking. Yeast cells were pelleted and resuspended in 30 to 50 μl PBS and immobilized on an agar pad prior to visualization. For tetracycline off strains, yeast cells were inoculated into 5 ml SD/−HIS supplemented with 300 μg/ml G418 and 50 μg/ml (PIK1 and STT4) or 300 μg/ml (MSS4) doxycycline (catalog no. 324385; Millipore) and incubated overnight at 30°C with shaking. Yeast cells were washed in TE buffer and resuspended in Gal/Raf/−HIS (to induce GFP fusion protein expression) with 300 μg/ml G418 and 50 μg/ml (PIK1 and STT4) or 300 μg/ml (MSS4) doxycycline. After overnight incubation, yeast cells were immobilized on agar pads as described above.

### Yeast lysis and Western blotting.

Yeast cells (*cdc25^ts^*) were inoculated into 5 ml SD/−Leu and incubated overnight at room temperature with shaking (250 rpm). To lyse, 1.5 ml of each culture was centrifuged at 14,000 rpm for 1 min. Each pellet was resuspended in 100 μl of 2.0 M LiAc and incubated on ice for 5 min. Samples were centrifuged at 14,000 rpm for 1 min to pellet, resuspended in 100 μl of 0.4 M NaOH, and incubated on ice for 5 min. Samples were pelleted as before, resuspended in 75 μl of 1× SDS Laemmli sample buffer, and boiled at 100°C for 5 min. Lysates were centrifuged at 14,000 rpm for 1 min to remove debris, separated by SDS-polyacrylamide gel electrophoresis, and transferred to polyvinylidene difluoride membrane for Western blotting. Fusion proteins were detected by rabbit anti-Ras (1:100), and equal loading was confirmed by detection with rabbit anti-glucose-6-phosphate dehydrogenase (anti-G6PDH) (1:10,000).

### Cell culture.

HeLa cells (ATCC CCL-2) were cultured in Dulbecco’s modified Eagle medium (DMEM) (Gibco) supplemented with 10% fetal bovine serum (FBS) (Gibco), 100 IU/ml penicillin, 100 μg/ml streptomycin, and 292 μg/ml l-glutamine (Corning). RAW267.4 macrophages (ATCC TIB-71) were cultured in RPMI 1640 (Gibco) supplemented with 10% heat-inactivated FBS, 100 IU/ml penicillin, 100 μg/ml streptomycin, 292 μg/ml l-glutamine, and 10 mM HEPES (HyClone).

To isolate primary human macrophages, 50 ml of blood from each donor was added to an equal volume of phosphate-buffered saline (PBS) and then separated by centrifugation over a Ficoll-Paque Plus (catalog no. GE17-1440-03; Sigma) gradient at 750 × *g* for 20 min with no break. The lymphocyte/monocyte layer was collected and incubated for 1 to 2 min with 1 ml ACK lysing buffer (catalog no. A10492-01; Gibco) to remove red blood cells. The cells were diluted to 50 ml in PBS and centrifuged 350 × *g* for 10 min at 4°C. The supernatant was removed, and the cells were washed in 25 ml PBS and pelleted at 160 × *g* for 15 min at 4°C. The cells were washed again in 25 ml PBS but centrifuged at 300 × *g* for 10 min at 4°C. This final pellet was resuspended in 5 to 10 ml of RPMI 1640 supplemented with 10% human AB serum (catalog no. 35-060-CI; Corning). To differentiate into macrophages, cells were cultured in RPMI 1640 supplemented with 10% human AB serum for at least 4 h to allow for attachment. Cells were washed in PBS, which was then replaced with RPMI 1640 plus 10% human AB serum and 50 ng/ml human macrophage colony-stimulating factor (M-CSF) (catalog no. 216-MC-025; R&D Systems) for 7 days with the medium being changed every 1 or 2 days.

### Antibodies.

To generate an antibody against native Mpt64, two rabbits were immunized with recombinant 6xHIS-tagged Mpt64ΔSP purified from E. coli in incomplete Freund’s adjuvant (Pacific Biosciences). The polyclonal rabbit antibody to antigen 85 (catalog no. NR-13800) and mouse anti-GroEL2 CS-44 (catalog no. NR-13813) are from BEI Resources. Chicken (ab94935), mouse (ab22683), and rabbit (ab2907) anticalreticulin were purchased from Abcam, and anti-GM130 (catalog no. 610822) was purchased from BD Biosciences. Mouse anti-Tom20 F-10 (sc-17764) and anti-β-Actin C4 (sc-47778) were purchased from Santa Cruz. Rabbit anti-Ras (catalog no. 3965S) and rabbit anti-glucose-6-phosphate dehydrogenase (anti-G6PDH) (catalog no. A9521) were purchased from Cell Signaling Technology and Sigma, respectively. Mouse anti-PMP70 CL2524 (MA5-31368), anti-CHOP 9C8 (MA1-250), and horseradish peroxidase (HRP)-conjugated secondary antibodies were purchased from Thermo Fisher Scientific. Alexa Fluor-conjugated secondary antibodies were from Life Technologies.

### Molecular biology.

Unless otherwise stated, all M. tuberculosis (Mtb) proteins were cloned from the BEI Resources Gateway Mtb ORF library using Gateway cloning technology (Life Technologies). The Mpt64 truncation mutants were PCR amplified (see Table S2 in the supplemental material) and cloned into pENTR (Life Technologies) prior to cloning into subsequent destination vectors.

10.1128/mSphere.00354-19.7 TABLE S2 Primers used in this study. DNA primers are listed in the 5′-to-3′ orientation. Primers paired together are labeled F for forward and R for reverse. Noncontiguous primer pairs are listed in the last column. Download Table S2, XLSX file, 0.005 MB. Copyright © 2019 Stamm et al. 2019 Stamm et al. This content is distributed under the terms of the Creative Commons Attribution 4.0 International license.

### hGH release assay and quantification.

HeLa cells were plated in 24-well plates to achieve approximately 50,000 cells/well 24 h prior to transfection. Cells were cotransfected with 1 μg hGH-CAD and 1 μg GFP-Mtb effector or GFP alone using FuGene 6 (Promega) per the manufacturer’s instructions. Cells were transfected 16 to 18 h at 37°C and 5% CO_2_. The transfection medium was then aspirated and replaced with DMEM containing 2 μM D/D Solubilizer (catalog no. 635054; Clontech) and incubated for 2 h at 37°C and 5% CO_2_. The plates were centrifuged at 1,500 rpm for 5 min to pellet debris, and the culture supernatants were saved at −80°C prior to human growth hormone (hGH) quantification.

Released hGH was quantified by ELISA (catalog no. 11585878001; Roche). Briefly, samples were thawed on ice, and 20 μl was transferred to each well containing 180 μl sample buffer (1:10). The plate was incubated for 1 h at 37°C, washed five times in 250 μl wash buffer, and incubated 1 h at 37°C with a polyclonal antibody to hGH conjugated to digoxigenin (anti-hGH-DIG). The plate was washed as described and incubated 1 h at 37°C with a polyclonal antibody to digoxigenin conjugated to peroxidase (anti-DIG-POD). The plate was washed and developed in peroxidase substrate (2,2'-azinobis [3-ethylbenzothiazoline-6-sulfonic acid]-diammonium salt). The absorbance was read on a Biotek plate reader at 405 nm.

### PIP strips membrane binding.

6xHIS-Mpt64_24-228 and 6xHIS-Mpt64_24-143 were purified by cobalt Talon affinity resin (Clontech). PIP strips membranes (catalog no. P23751; Invitrogen) were blocked for 1 h at room temperature in 3% fatty acid-free bovine serum albumin (BSA) (catalog no. A7030; Sigma) in TBST. Mpt64_24-228 or Mpt64_24-143 was diluted to 1.5 μg/ml in 3 ml of 3% fatty acid-free BSA and incubated with the PIP strips for 3 h at room temperature with agitation. Membranes were washed three times in 3% fatty acid-free BSA prior to incubation with anti-Mpt64 or preimmune serum (1:3,000) overnight at 4°C with agitation. Membranes were washed three times in 3% fatty acid-free BSA and then incubated with HRP-conjugated donkey anti-rabbit (1:2,000) for 30 min at room temperature. Membranes were washed three times before detection of Mpt64 lipid interactions by chemiluminescence.

### Transfection and colocalization of MSPs in HeLa cells.

HeLa cells were transfected overnight with GFP fusion proteins using FuGene 6 transfection reagent (Roche). Cells were fixed in 4% paraformaldehyde (PFA) for 15 min, washed in PBS, and permeabilized in 0.25% Triton X-100 for 3 min at room temperature or in 100% methanol for 10 min at −20°C when using Tom20 antibody. Cells were stained with organelle-specific antibodies for 1 h at room temperature. Antibodies were visualized by secondary antibodies conjugated to Alexa Fluor 594. Cells were mounted in ProLong Gold plus DAPI (catalog no. P36931; Invitrogen), and z-stacks were collected on an AxioImager M2 microscope (Zeiss).

### Detection of CHOP accumulation in macrophages.

RAW267.4 cells stably expressing Mpt64ΔSP under a cytomegalovirus (CMV) promoter or control cells transduced with an empty lentivirus were seeded in 12-well plates at 5 × 10^5^ cells/well. To induce the unfolded protein response (UPR), the culture medium was replaced with media supplemented with 50 nM thapsigargin (catalog no. T9033; Sigma) or an equal volume of vehicle (dimethyl sulfoxide [DMSO]), and cells were incubated for 4 h. The cells were washed twice in PBS and lysed in ice-cold RIPA buffer supplemented with protease inhibitor tablets (catalog no. 11836153001; Roche). Lysates (15 to 25 μg) were separated by SDS-polyacrylamide gel electrophoresis and transferred to a polyvinylidene difluoride membrane for Western blotting. Accumulation of CCAAT enhancer-binding protein homologous protein (CHOP) was detected by mouse anti-CHOP (1:2,000), and band density was normalized to bands detected by the loading control mouse antiactin (1:3,000).

### Infection and colocalization of Mpt64 in macrophages.

Bacteria were washed repeatedly in PBS and then sonicated to create a single-cell suspension. RAW267.4 cells were infected in DMEM plus 10% horse serum (catalog no. 26050088; Invitrogen) at a multiplicity of infection (MOI) of 20:1 with mycobacteria expressing mCherry. Cells were centrifuged at 1,500 rpm for 10 min to permit bacterial attachment and then allowed to phagocytose for 1.5 h at 37°C and 5% CO_2_. Cells were fixed after 4 h postinfection in 4% PFA for 60 min. Cells were permeabilized in 0.25% Triton X-100 for 3 min at room temperature and then blocked in 5% normal donkey serum (Sigma). Mpt64 was detected with rabbit anti-Mpt64 antibody (1:500) and an HRP-conjugated goat anti-rabbit secondary antibody (1:1,000; Santa Cruz). Antibody signal was amplified by the addition of biotinylated tyramide (1:50; PerkinElmer) with detection by Alexa Fluor 488-conjugated streptavidin (1:250; Jackson Immunoresearch) or cyanine 5 tyramide (1:50; PerkinElmer). Z-stack slices were acquired with an AxioImager M2 microscope (Zeiss).

Primary human macrophages were infected in RPMI 1640 plus 10% human AB serum at an MOI of 10:1 with mycobacteria expressing mCherry for 2 h at 37°C and 5% CO_2_ to allow for phagocytosis. Cells were washed and fixed at 4 h postinfection in 4% PFA for 45 to 60 min. Cells were permeabilized in 100% ice-cold methanol for 10 min at −20°C and blocked in 5% normal goat serum (Sigma). Mpt64 was detected with rabbit anti-Mpt64 antibody (1:500) and an HRP-conjugated donkey anti-rabbit secondary antibody (1:500; Thermo Fisher Scientific) followed by amplification with cyanine 5 tyramide (1:50; PerkinElmer). Colocalization of Mpt64 with the endoplasmic reticulum (ER) was detected with chicken anticalreticulin (1:100), followed by goat anti-chicken-Alexa Fluor 488 (Abcam).

### Macrophage infections for CFU.

Primary human macrophages were seeded in low-evaporation 24-well plates at approximately 5 × 10^5^ cells/well. Bacteria were washed repeatedly in PBS and then sonicated to create a single-cell suspension. Macrophages were infected in RPMI 1640 plus 10% human AB serum at a MOI of 0.1:1. Cells were centrifuged at 1,500 rpm for 10 min to permit bacterial attachment and then allowed to phagocytose for 15 min at 37°C and 5% CO_2_. The cells were washed in PBS and then replaced with RPMI 1640 plus 10% human AB serum, and cells were washed every day between time points. The cells were lysed at time zero and subsequent time points in 500 μl of0.5% Triton X-100 in PBS. Serial dilutions were plated on Middlebrook 7H11 plates, and colonies were enumerated after 2 to 3 weeks.

### Construction of the Mtb *mpt64* deletion mutant and complementation.

An in-frame *mpt64* deletion in Mtb was made using mycobacteriophage as previously described (112). Briefly, 500 bp 5′ to the *mpt64* start codon and 500 bp 3′ to the *mpt64* stop codon were amplified from Erdman genomic DNA (Table S2) and sequentially cloned into the multiple cloning sites of pMSG360HYG. This vector was linearized with AflII and DraI (catalog no. R0520 and R0129; New England Biolabs [NEB]) and transformed into E. coli EL350/phAE87 by electroporation. Phagemid DNA was isolated from pooled colonies and transformed into Mycobacterium smegmatis by electroporation. Plaques were isolated and pooled from M. smegmatis lawns, and high-titer phage was produced. Log-phase M. tuberculosis Erdman was transduced with phage at 42°C for 4 h. Mutants were selected on 7H11 plus hygromycin (100 μg/ml). Wild-type *mpt64* strain and a *mpt64* strain lacking its secretion signal were cloned into an integrating vector containing a constitutive promoter (pMV306_MSP), conferring zeocin resistance. The MtbΔ*mpt64* was transformed by electroporation, and complements were selected on 7H11 plus zeocin (25 μg/ml).

To confirm expression and secretion of Mpt64 complements, Mtb strains were grown to late-log phase and pelleted by centrifugation. The culture supernatants were saved and passed twice through 0.22-μm filters. Bacterial pellets were boiled for 30 min in lysis buffer (50 mM Tris [pH 7.4], 150 mM NaCl) supplemented with Complete Mini protease inhibitor and then subjected to bead beating to lyse the cells. Protein content in lysates was determined by Bradford assay. Mpt64 expression in the lysates and culture supernatants was detected by Western blotting using a rabbit polyclonal antibody to Mpt64 (1:10,000). Equal loading of samples in the lysates and supernatants was confirmed by Western blotting with anti-GroEL2 (1:500) and anti-antigen 85 (1:1,000), respectively.

### Mouse infections.

Female BALB/c mice (The Jackson Laboratory) were infected via aerosol as described previously (149). Briefly, mid-log-phase Mtb bacteria were washed in PBS repeatedly and then sonicated to create a single-cell suspension. Bacteria were resuspended to yield an OD_600_ of 0.1 in PBS. This suspension was transferred to the nebulizer of a GlassCol aerosolization chamber calibrated to infect mice with ∼100 bacteria per animal. On the day of infection, whole lungs were collected from five mice per group, homogenized, and plated on Middlebrook 7H11 to determine the initial inoculum. At subsequent time points, the left lung, spleen, and left lobe of the liver were used to determine CFU, while the right lung was insufflated with 10% neutral buffered formalin for histopathology.

### Lysozyme pulldown.

E. coli lysates containing six-histidine (6xHIS)-tagged Mpt64 or an unrelated protein Cor were incubated with cobalt affinity resin (Talon; Clontech) to bind histidine-tagged proteins. After extensive washing, 1 mg/ml of either hen egg white lysozyme (catalog no. BP535; Fisher Scientific) or human lysozyme (catalog no. L1667; Sigma) was forced to flow over the immobilized beads and incubated for 5 min. The beads were washed two more times before proteins were eluted with 300 mM imidazole.

### Mtb genomic DNA isolation.

Late-exponential-phase Mtb was collected by centrifugation and washed once in PBS. Pellets were boiled for 20 to 30 min to sterilize. Pellets were washed once in GTE (25 mM Tris [pH 8.0], 10 mM EDTA, 50 mM glucose) and incubated overnight in lysozyme solution (10 mg/ml in GTE) at 37°C. Samples were incubated in 10% SDS and 10 mg/ml proteinase K for 40 min at 55°C, followed by incubation in NaCl and CTAB (2.4 M NaCl, 274 mM cetrimonium bromide [catalog no. H9151; Sigma]) at 60°C for 10 min. Genomic DNA was then isolated using a phenol-chloroform extraction, followed by ethanol precipitation.

### Extraction of apolar lipids and PDIM analysis.

Log-phase Mtb or MtbΔ*mpt64* were synchronized to OD_600_ of 0.2 in Middlebrook 7H9 supplemented with 0.01% Tween 80 and grown for 24 h. Bacteria were collected by centrifugation at 1,600 × *g* for 10 min, resuspended in 1 ml of 15% isopropanol, and transferred to a glass tube containing 5 ml chloroform-methanol (17:1, vol/vol), and incubated 24 h at room temperature. Samples were centrifuged at 1,600 × *g* for 5 min, and the apolar lipids were collected from the bottom organic layer and dried. Apolar lipids were resuspended in 1.5 ml of 100% methanol. Tween 80 was removed by the addition of cobalt/thiocyanate solution and vortexed. The remaining lipids were extracted by the addition of 4 ml hexane. After centrifugation, the organic layer was saved and the aqueous layer was reextracted with 4 ml hexane. Both hexane fractions were combined, dried, and resuspended in 1 ml chloroform-methanol (2:1, vol/vol). The phthiocerol dimycocerosate (PDIM) standard was similarly resuspended. The PDIM standard or apolar lipids extracted from Mtb or MtbΔ*mpt64* were infused into an AbSciex TripleTOF 5600/5600+ mass spectrometer. Samples were analyzed in the positive mode.

### Statistical analysis.

Statistical analysis was performed using GraphPad Prism software. For *in vitro* studies, one-tailed analysis of variance (ANOVA) tests were used for experiments with multiple comparisons using Dunnett’s test. For experiments with single comparisons, two-tailed unpaired Student’s *t* test was used. For experiments containing samples with nonnormal distributions such as *in vivo* CFU measurements and area of lung inflammation, the Kruskal-Wallis nonparametric test was used with Dunn’s correction for multiple comparison. Analysis of survival studies was performed by Kaplan-Meier test.

### Ethics statement.

Primary human macrophages were isolated from buffy coats from anonymous donors provided by a local blood bank (Carter Bloodcare). This study was reviewed by the University of Texas (UT) Southwestern Institutional Review Board and deemed to be exempt.

Animal experiments were reviewed and approved by the Institutional Animal Care and Use Committee at the University of Texas Southwestern (protocol 2017-102086) and followed the eighth edition of the *Guide for the Care and Use of Laboratory Animals* (150). The University of Texas Southwestern is accredited by the American Association for Accreditation of Laboratory Animal Care (AAALAC).
