# Supplementary figures and images for: Screening Mycobacterium tuberculosis Secreted Proteins Identifies Mpt64 as a Eukaryotic Membrane-Binding Bacterial Effector
Source: mSphere. 2019 Jun 5;4(3):e00354-19. doi: 10.1128/mSphere.00354-19 (PMC6553557; doi:10.1128/mSphere.00354-19)

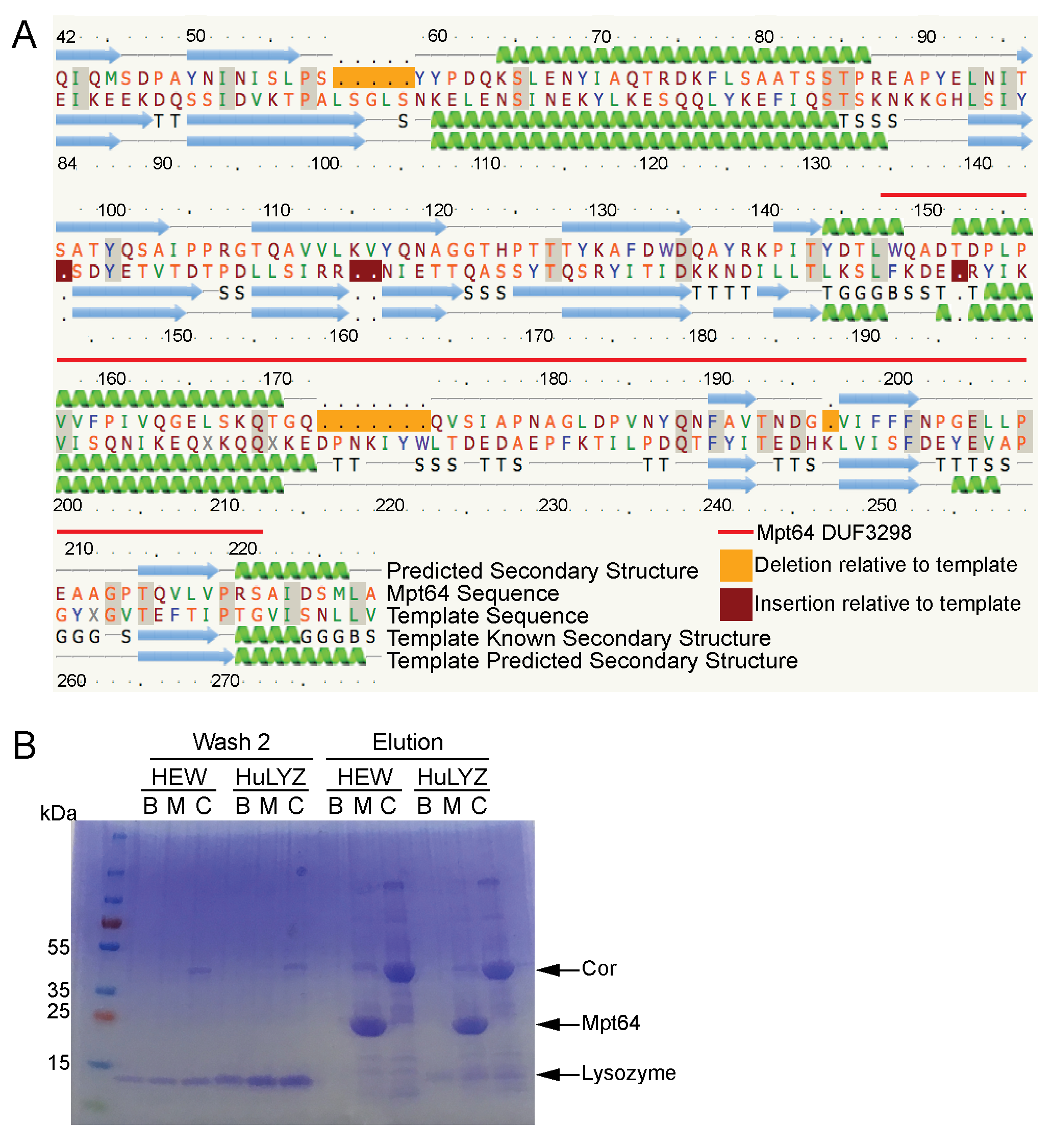

Supplement: FIG S1 [file mSphere.00354-19-sf001.tif]

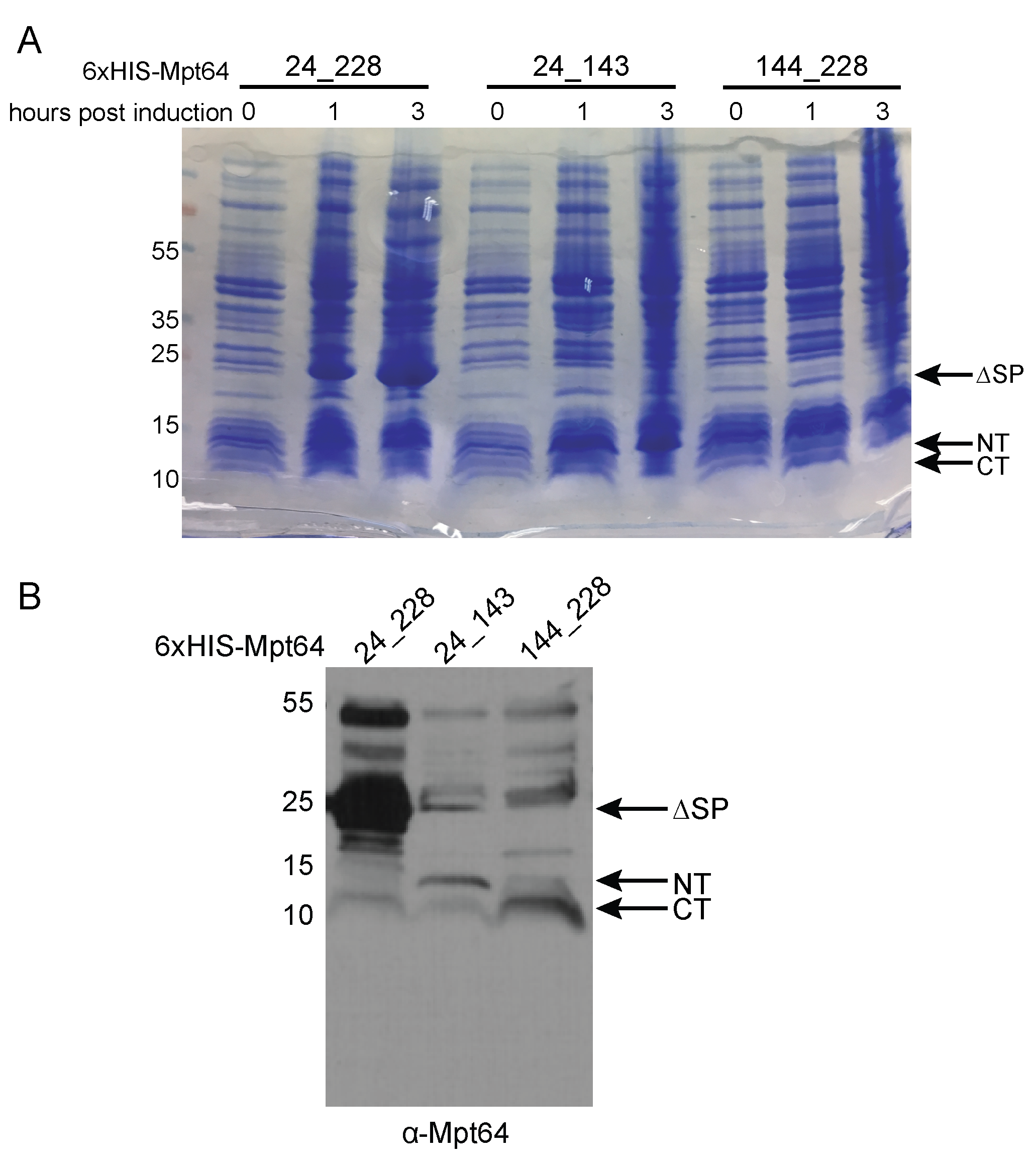

Supplement: FIG S2 [file mSphere.00354-19-sf002.tif]

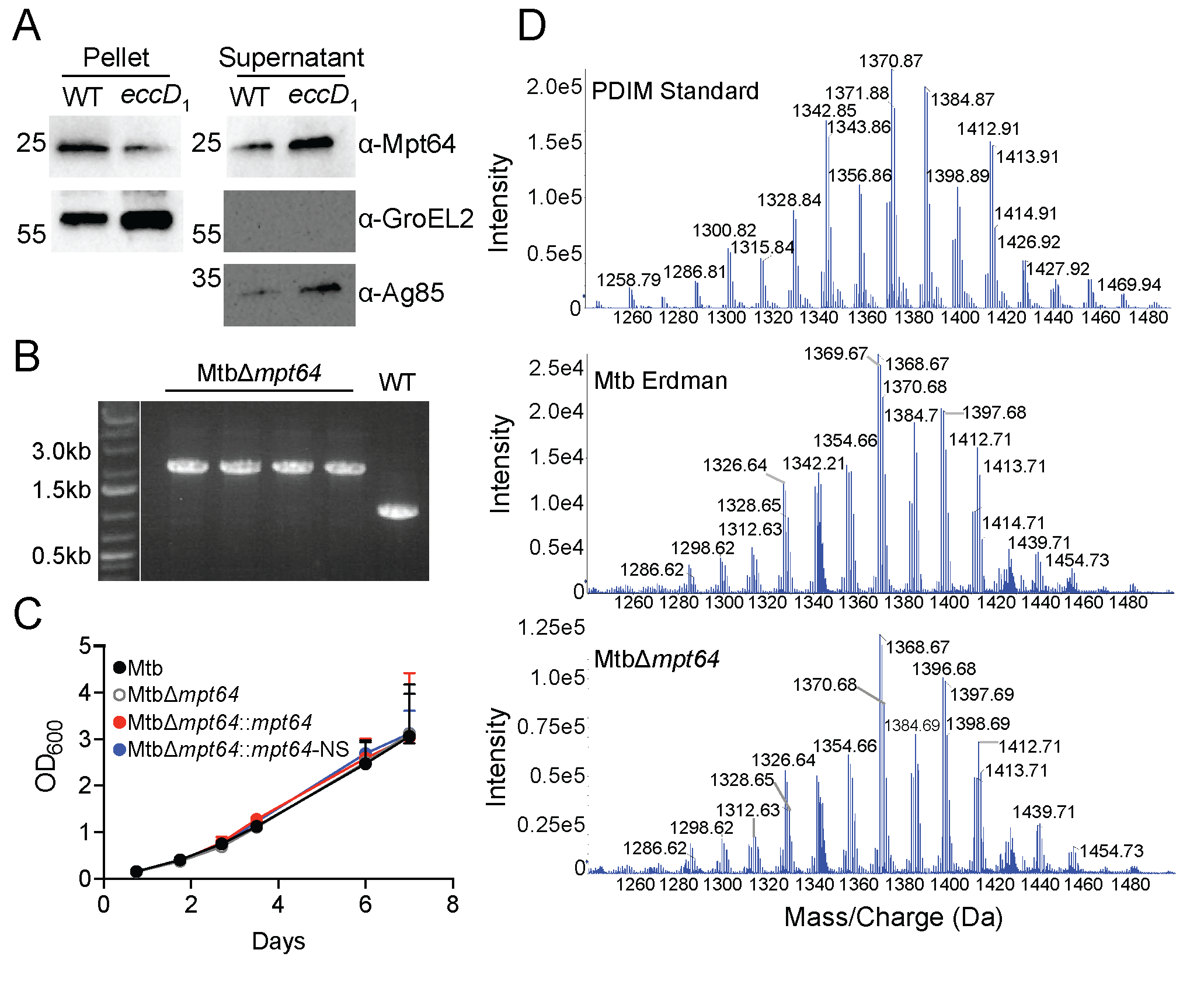

Supplement: FIG S3 [file mSphere.00354-19-sf003.tif]

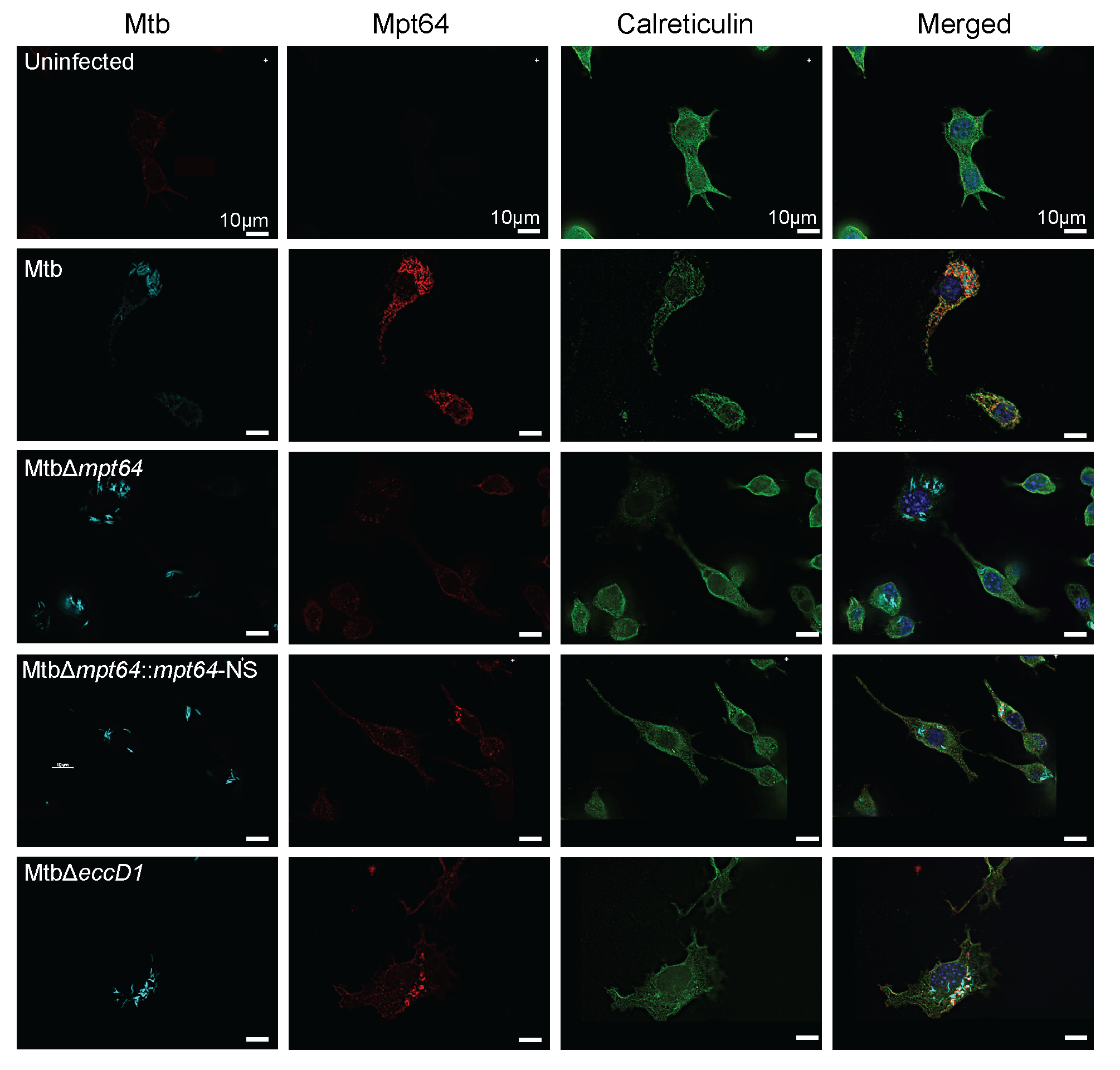

Supplement: FIG S4 [file mSphere.00354-19-sf004.tif]

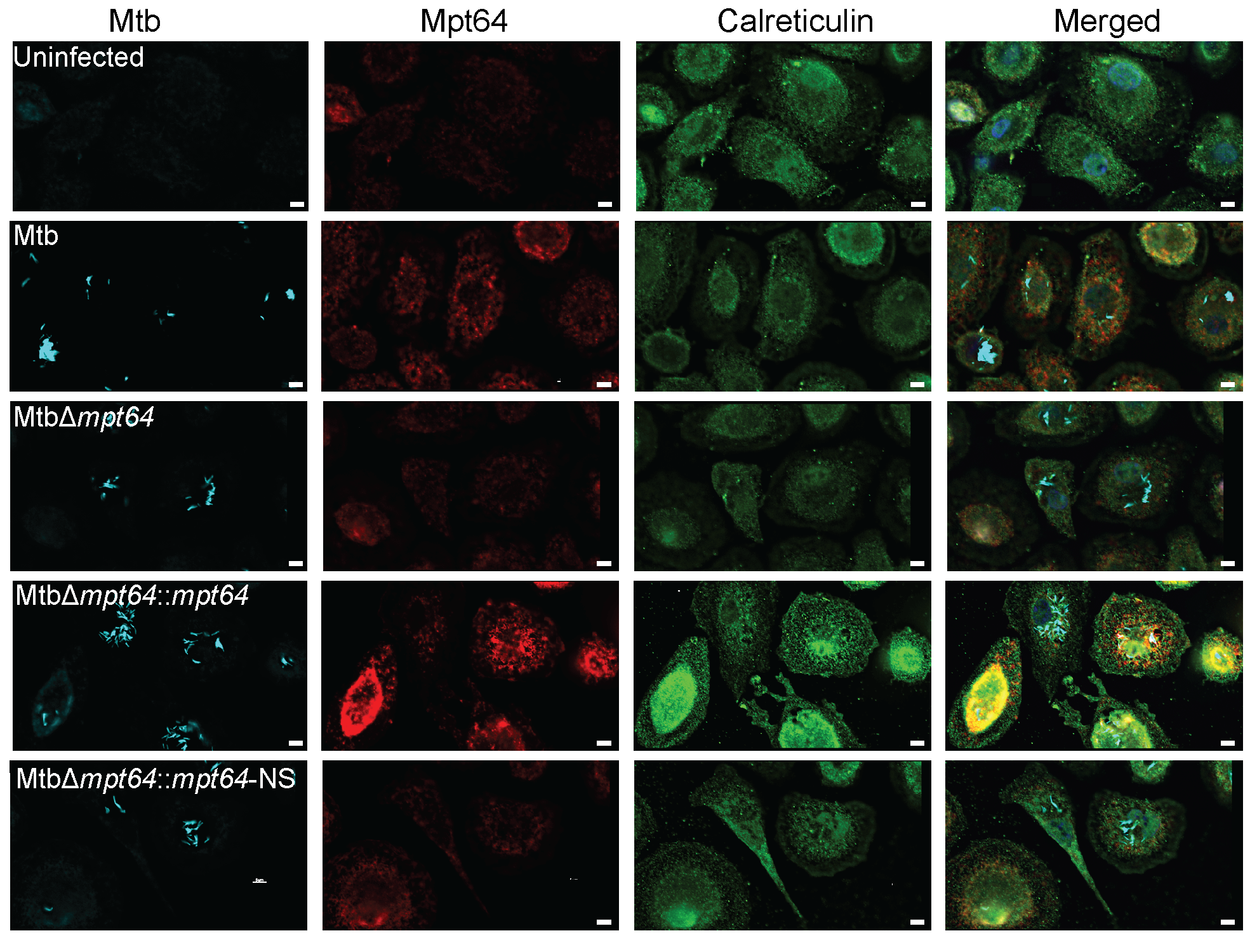

Supplement: FIG S5 [file mSphere.00354-19-sf005.tif]
